# Sensing of joint and spinal bending or stretching via a retractable and wearable badge reel

**DOI:** 10.1038/s41467-021-23207-8

**Published:** 2021-05-19

**Authors:** Chengyu Li, Di Liu, Chaoqun Xu, Ziming Wang, Sheng Shu, Zhuoran Sun, Wei Tang, Zhong Lin Wang

**Affiliations:** 1 CAS Center for Excellence in Nanoscience, Beijing Institute of Nanoenergy and Nanosystems, Chinese Academy of Sciences, Beijing, China; 2 Center on Nanoenergy Research, School of Physical Science & Technology, Guangxi University, Nanning, China; 3 School of Nanoscience and Technology, University of Chinese Academy of Sciences, Beijing, China; 4 Department of Orthopedic, Peking University Third Hospital, Beijing, China; 5 School of Materials Science and Engineering, Georgia Institute of Technology, Atlanta, GA USA; 6 CUSPEA Institute of Technology, Wenzhou, Zhejiang China

**Keywords:** Devices for energy harvesting, Sensors and biosensors, Nanoscale devices

## Abstract

Human motions, such as joint/spinal bending or stretching, often contain information that is useful for orthopedic/neural disease diagnosis, rehabilitation, and prevention. Here, we show a badge-reel-like stretch sensing device with a grating-structured triboelectric nanogenerator exhibiting a stretching sensitivity of 8 V mm^−1^, a minimum resolution of 0.6 mm, a low hysteresis, and a high durability (over 120 thousand cycles). Experimental and theoretical investigations are performed to define the key features of the device. Studies from human natural daily activities and exercise demonstrate the functionality of the sensor for real-time recording of knee/arm bending, neck/waist twisting, and so on. We also used the device in a spinal laboratory, monitoring human subjects’ spine motions, and validated the measurements using the commercial inclinometer and hunchback instrument. We anticipate that the lightweight, precise and durable stretch sensor applied to spinal monitoring could help mitigate the risk of long-term abnormal postural habits induced diseases.

## Abstract (web summary)

Human motions often contain information that is useful for orthopedic/neural disease diagnosis, rehabilitation, and prevention. Here, the authors show a badge-reel-like stretch sensing device with a grating-structured triboelectric nanogenerator for joints/spine bending or stretching sensing.

## Introduction

Human motions, such as bending and stretching, with various amplitudes and velocities ranging from slight movement to full-body kinematics, contain diverse and important physiological health information^1,2^, and this physiological health information is often closely or potentially related to diseases, for example, the freezing of gait, a paroxysmal block of movement, often takes place in the advanced stage of Parkinson’s disease^3,4^; muscle aches and weakness in limbs, leading to movement disorders, might suggest early rheumatism^5,6^. Additionally, office people now sit for long periods of time everyday^7^. Maintaining such one single posture for a long time might probably lead to disorders in the spine^8^, as well as fatigue for their body, regarded as the major factors resulting in a significant increase in the risk of back pain^9,10^. According to recent researches, over 60% of Americans and 50% of Europeans are about to experience back and neck pain at some point in their lives^11,12^. Worse, that time tends to come in advance.

Traditional cameras^13^ and inertial measurement units^14^ (IMUs) can be used for monitoring human motions and postures potentially^15^. However, the former is not a wearable technique. When the subject moves from one place to another, out of the camera’s view, the monitoring will be interrupted. In addition, the IMUs are mainly based on point measurement, requiring complicated body parameters for post-computational modeling to calculate out the motions, which is indirect. As we know, during the inertial measurement, misalignment error grows as a function of time, which will reduce the sensing system’s precision, and thus extra correction is required from time to time. Therefore, direct, conformal, and wearable sensing techniques are in demand. Recent advances in soft electronics put forward flexible and stretchable functional sensors^16–18^, mainly based on piezoresistive^19^, capacitive^20^, and piezoelectric architectures^21^. They are continuous and conformal on human bodies, exhibiting high stretchability and high performance^22–24^. However, as for the soft piezoresistive and capacitive devices, challenges, resulting from materials^17,20,22^, still remain, such as hysteresis, robustness, and environmental interferences, whereas, devices with rigid parts and soft connections might suffer from nonlinearity. The newly developed triboelectric nanogenerator (TENG)^25^ combines flexible materials/substrates that make the overall device wearable, with sophisticated circuit fabrication engineering^26^ that ensures the device work stable, and demonstrates its advances in either kinetic energy converting^27^ or self-powered motion sensing^28^.

Here, we describe a badge-reel-like stretch sensor, based on a grating-structured triboelectric nanogenerator (TENG)^29,30^. It stretches and contracts, synchronously with human subject bending and stretching, exhibiting a high sensitivity of 8 V mm^−1^, a minimum resolution of 0.6 mm, excellent robustness (over 120-thousand stretching cycles), and a low hysteresis. We used it to record joint motions, such as knee/arm bending, neck/waist twisting, demonstrating its functionality for real-time monitoring. Furthermore, we attached it along the spine, including S1–L1, L1/T12–C7, C1–C7, and S1–C7 (C, T, L, and S represent the cervical, thoracic, lumbar, and sacrum segments of the spine, respectively, and the numbers stand for the corresponding stacked bones), to detect the spinal shape change, presenting its potential application in daily spinal monitoring.

## Results

### Device and structural design

**Fig. 1:**
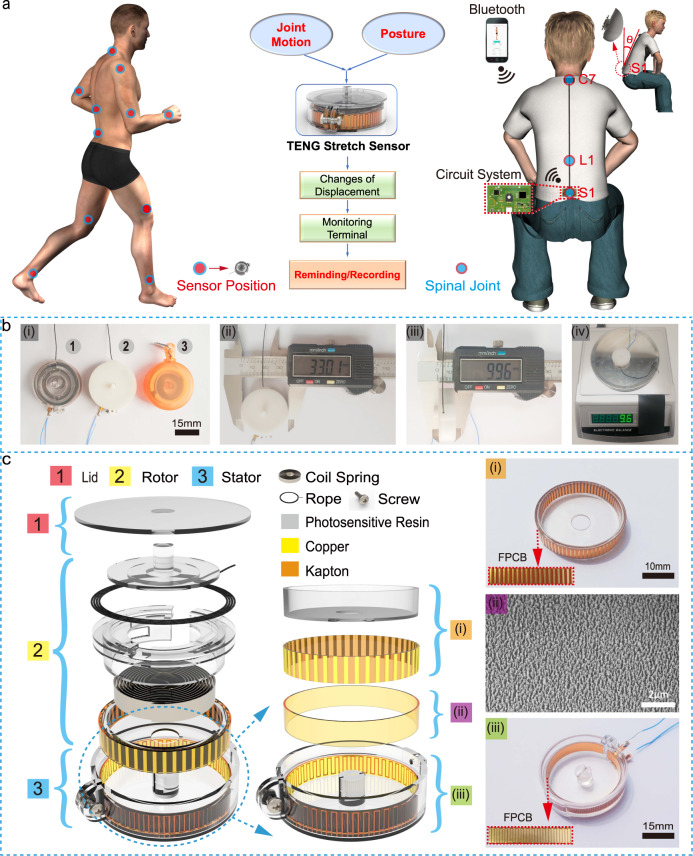
Concept illustrations, schematic diagrams, photographs, and exploded view of the stretch sensor. **a** Illustration of the thin, lightweight, and wearable stretch sensor placed around the full body to monitor the joint and spinal motions, with continuous, wireless monitoring ability. **b** Images from a top view showing (i) the representative stretch sensors made up of two different 3D printing materials, i.e., photosensitive resin (1) and white resin material (2), and (3) displays a commercial retractable badge reel. In addition, the diameter (ii) and height (iii) of the sensor are shown. **c** Exploded view of the stretch sensor, consisting of (1) lid, (2) rotor, and (3) stator, and enlarged images: (i) the rotor electrode, (ii) etched nanorods on the surface of Kapton film, and (iii) the stator electrode.

By combining a retractable badge reel and the grating-structured triboelectric nanogenerator (TENG), a small-volume and high-precision stretch sensor has been developed for wearable and real-time monitoring human motions (Fig. 1a). The sensor retains a simple operation principle of a retractable reel: with two ends placed on the subject body, it extends or contracts, as the human subject bends or strengthens. The as-generated electrical signals from TENG are obtained, processed, and then transmitted via Bluetooth to the monitoring terminal, e.g., a cell phone.

Figure 1bi shows the image of three fabricated devices, encapsulated with various three-dimensional (3D) printing boxes. The overall device takes a diameter of 33 mm, a thickness of 10 mm, and a weight of 9.6 g (Fig. 1bii–iv). Due to the small volume and lightweight, it is able to be taped on the skin, or mounted in some vests.

Figure 1c shows an exploded view of the device. The construction involves (1) the lid, (2) the rotor, and (3) the stator, coaxially assembled. The rotor is mainly made up of a coil spring (see Supplementary Fig. 2 for the elastic coefficient curves of three different coil springs) and a group of grating electrodes (Cu, ~35-μm thick; height, 5 mm; width, 0.5 mm; spacing, 0.1 mm), while the stator includes two groups of complementary grating electrodes (Cu, ~35-μm thick; spacing, 0.1 mm), covered by Kapton (~35-μm thick), with its surface ion-etched in order to enhance the output performance (Fig. 1cii). The grating electrodes on the rotor and stator, and the Kapton film, compose into a freestanding TENG. A screw is used to adjust the gap between the rotor and stator, which can thereby adjust the sensor’s output performance. A detailed description of the overall fabrication procedure is available in Supplementary Fig. 1 and Methods.

### Working principle and electrical characteristics

**Fig. 2:**
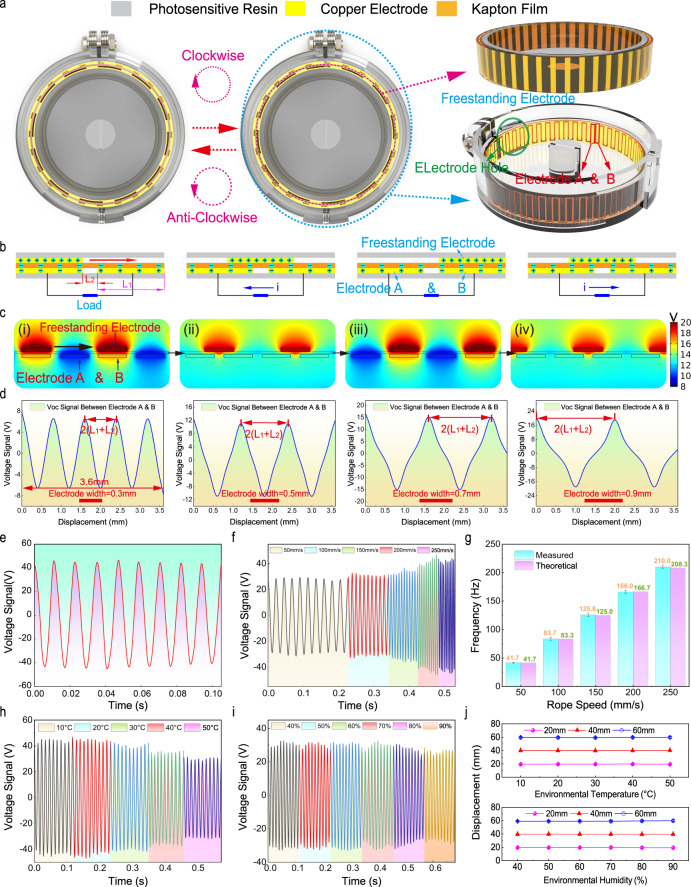
The working principle, output performance, and characteristics of temperature and humidity of the device. **a** A top view of the stretch sensor when rotating clockwise and anticlockwise, and the right side of the image indicates the detail of the device’s 3D structure. **b** Schematic diagram of the working mechanism of the stretch sensor based on freestanding triboelectric nanogenerators (TENG). **c** Potential simulation of one working cycle by FEA with 0.3-mm electrode width, and (**d**) the simulated results of the open-circuit voltage with different electrode widths 0.3, 0.5, 0.7, and 0.9 mm. **e** The basic electrical output characteristic diagram of the stretch sensor with 0.5-mm electrode widths. **f** Voltage signals with different velocities from 50 to 250 mm s^−1^ under a given displacement (36 mm) and **g** represents the theoretical and measured frequency corresponding to different velocities. **h**–**i** The output performance of the sensor at different environmental temperatures and humidity, and its corresponding displacement accuracy is shown in **j**. All error bars represent standard deviation based on ten replicate data under the same test condition. Source data are provided as a Source Data file.

Figure 2a displays the top view of the rotor electrodes rotating clockwise relative to the stator electrodes, where the left and middle images show respectively the state when the rotor’s electrodes coincide with the electrodes A or B on the stator (more detail for the working mechanism of the sensor, see Supplementary Fig. 3). The right image shows the rotor and stator’s 3D structural schematics, from which we can see the complementary grating electrodes A and B located on the stator, and the freestanding electrodes located on the rotor. Via the electrode hole on the backside, electrodes A and B can be connected with the external measurement circuit.

Figure 2b shows the working mechanism of the stretch sensor based on the triboelectric nanogenerator (TENG). During rotating, the freestanding electrodes rub with the Kapton film, inducing electrification^31,32^. Due to the charge-transferring law, the charge density on the rotor electrode is about two times that on the Kapton. In the initial state (the rotor electrodes align with electrode A on the stator), induced charges accumulate on electrodes A and B with the same charge amount but opposite polarity. As the rotor slides, free electrons keep flowing from electrode A to electrode B via the external circuit, until the rotor reaches the final state where the charge on both electrodes is the same in amount but reversed in polarity. The electron flow is driven by electrostatic force in order to reach an electrostatic equilibrium. As the rotor keeps sliding, the electrons will periodically flow forth and back (the working principle of reverse rotation is shown in Supplementary Fig. 5a).

Figure 2c illustrates the finite element analysis (FEA, by COMSOL Multiphysics) results of the open-circuit potential distribution on electrodes A and B during rotation with the electrode width of 0.3 mm. Figure 2d plots out the voltage between the electrodes A and B (with various electrode widths), as the rotor slides. It exhibits a periodic voltage waveform. As the electrode width increases, the peak-to-peak value rises. More FEA results can be found in Supplementary Fig. 5 and Supplementary Movie 1. In theory, the variation trend of the *V*_*oc*_ can be approximately expressed as follows (detailed derivation is available in Supplementary Note 1):

1 $${V}_{OC}(x)=\frac{d\cdot \sigma }{{\varepsilon }_{0}{\varepsilon }_{r}}\cdot \left(\frac{{L}_{1}-x}{x}-\frac{x}{{L}_{1}-x}\right)$$

where *L*_*1*_ represents the electrode width, *L*_*2*_ the electrode gap (considering that the value of *L*_*2*_ is negligible compared to that of *L*_*1*_, *L*_*2*_ can be ignored), *d* the dielectric material’s thickness, *σ* the tribocharge density, *ε*_*0*_ vacuum permittivity, *ε*_*r*_ the dielectric material’s relative permittivity, *x* the displacement of the freestanding electrode. The equation shows that when the electrode width *L*_*1*_ increases, the voltage will rise, consistent with the FEA simulations. It is also worth noting that (Fig. 2d), when the rotor slides over a distance of *L*_*1*_ + *L*_*2*_, *V*_*oc*_ reverses, shifting from the peak to the valley, or the valley to the peak, meaning a half-period. Therefore, the overall displacement can be figured out by counting the number of half a period *N*, and *L*_*1*_ + *L*_*2*_ stands for the minimum resolution. In addition, the voltage signal’s frequency can be expressed as the following formula:

2 $$f=\frac{v}{2({L}_{1}+{L}_{2})}$$

where *v* represents the sliding velocity of the rotor (the freestanding electrode). Subsequently, we used an oscilloscope (DSO2014A Keysight, USA) to examine the sensor’s performance. Figure 2e displays an output of a device with its electrode width of 0.5 mm (more details are available in Supplementary Movie 2). It exhibits a periodic waveform, with a peak-to-peak value ~80 V. As the sliding velocity increases, the output frequency becomes higher, as well as the output current, due to that with a given transferred charge amount, the velocity rises, the transferring time shortens (Fig. 2f). It is worth noting that we employed the oscilloscope, which is equivalent to a 100-MΩ resistor, to measure the voltage, and thus, the voltage value increases as the current increases. Subsequently, as a comparison, we plot the measured frequency and theoretical value (calculated by Eq. (2)) in Fig. 2g, which are consistent well, indicating a high fabrication precision. For more output performance tests of electrodes with different electrode widths, see Supplementary Fig. 6.

Moreover, as a wearable sensor, the electrical output of the device might be inevitable to be affected by environmental temperature and humidity. We studied this and plot results in Fig. 2h, i. It is found that the output voltage will decrease slightly as the temperature rises (Fig. 2h). And the increase of humidity has a bare significant effect on the performance, due to the protection of the encapsulation (Fig. 2i). However, the displacement achieved by our device is determined by the half-period number *N* and the structural length *L*_*1*_ + *L*_*2*_, as discussed above. This approach apparently avoids the output value variation owing to the environmental interferences. As we can see in Fig. 2j, for displacements of 20, 40, and 60 mm, under different temperatures and humidity, the sensor delivers precise displacement measures.

### Full-body bending monitoring by the stretch sensor

**Fig. 3:**
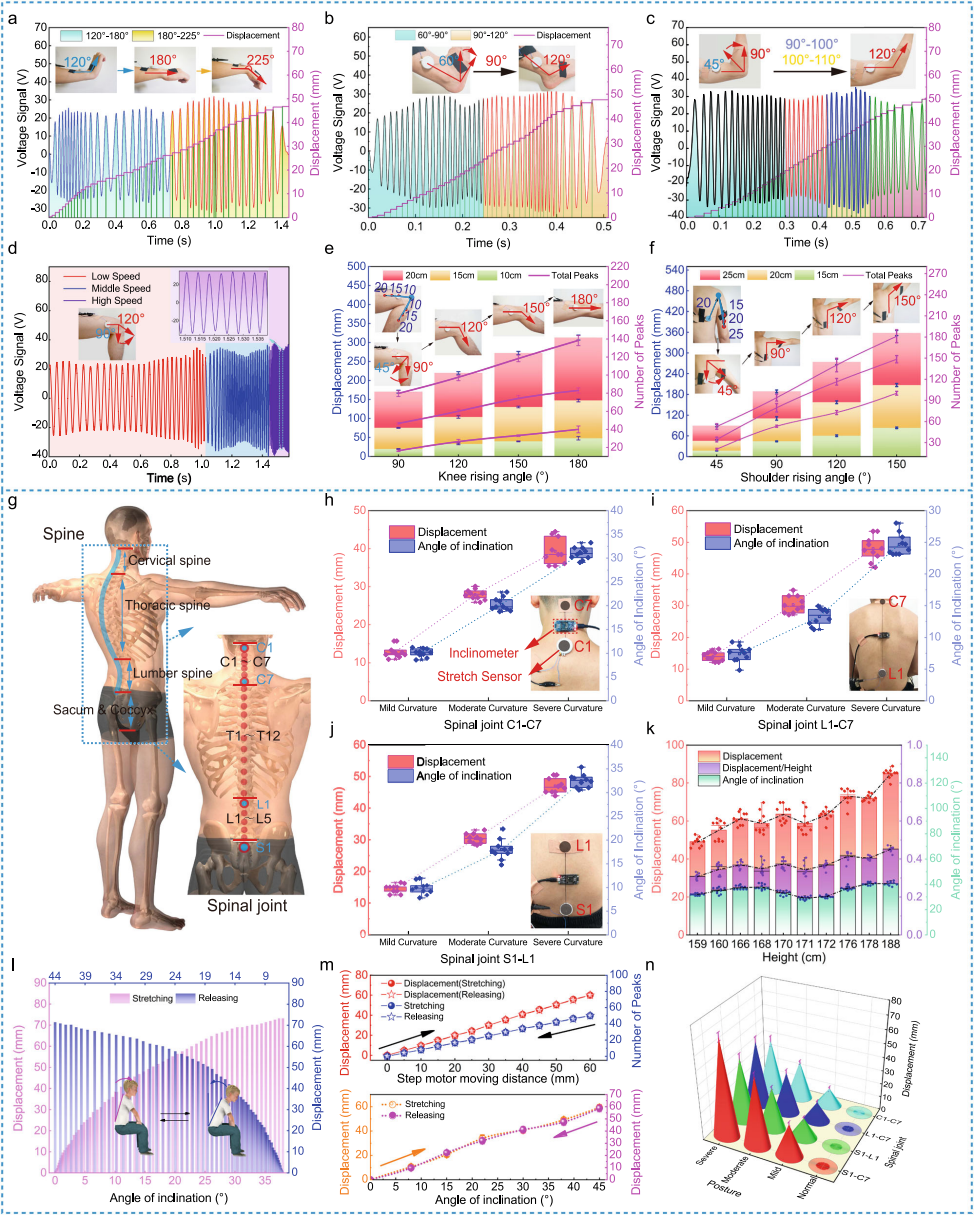
Detection of multijoint movements and spinal bending using the stretch sensor. **a**–**c** The measurement of dynamic wrist/ankle/elbow bending via the stretch sensor attached on the body, respectively. **d** The voltage output signal of knee bending at different speeds. **e**, **f** Comparisons of knee/shoulder’s angle variation with the stretching displacement. **g** Schematic illustration of a human spine model with devices’ potential fixed positions marked with blue dots. **h**–**j** Detection of stretching displacement of cervical segment C1–C7, thoracic segment L1–C7, and lumbar segment S1–L1 at different bending degrees included mild curvature, moderate curvature, and severe curvature. The photographs of the device mounting on the human body are also inserted. **k** The relationship between the bending angle and the displacement (unnormalized and normalized) measured by the stretch sensor. **l** The relationship between the bending angle and the displacement when bending up and down (the sensor is fixed on spinal process S1–C7 and the volunteer is 178-cm high). **m** The hysteresis characteristics of the stretch sensor measured by a linear motor (the image above) and a volunteer (the image below) respectively, showing almost no hysteresis in a cycle of stretching and releasing. **n** The stretch sensor was fixed to different locations of spinous processes (C1–C7, L1–C7, S1–L1, and S1–C7), showing different displacement variations. All error bars represent standard deviation based on ten replicate data, including displacement, angle, and the number of peaks. Source data are provided as a Source Data file.

Figure 3a–f shows the application of using the stretch sensor to monitor motions of the human wrist, ankle, elbow, knee, and shoulder. As shown in Fig. 3a, the stretch sensor is fixed between the forearm and the hand, across the wrist, to record the voltage response under the wrist’s stretching/releasing. With the wrist bending from 120° to 225°, the sensor outputs continuous voltage signals, measured by the oscilloscope. The peak value is ~20–30 V, determined by the bending speed^33^, while the stretching displacement increased step by step, with one step meaning 0.6 mm. Similarly, bending motions of the ankle (Fig. 3b) and the elbow (Fig. 3c and Supplementary Movie 3) can also be measured. Furthermore, as shown in Fig. 3d, the sensor is fixed between the thigh and the calf, and we examined the sensor performance under a fixed angle and various bending speeds (for more details, see Supplementary Movie 3). It is indicated that high speed will lead to high voltage and frequency, but the same peak number and displacement value. In addition, Fig. 3e suggests excellent linearity between the displacement and the bending angle. Afterward, we placed the sensor in different positions on the thigh, resulting in different initial lengths. The measured results show that the farther the sensor is away from the joint rotation point, the larger the displacement is, as well as the peak number. Similar results can be found in the test on the shoulder (Fig. 3f).

Current clinical investigations into the spine are lacking of wearable and real-time technologies and mainly based on X-ray film, which is time-consuming and harmful. IMU technique could be applied, however, it requires complicated body parameters to build the individual body model, and the measuring is indirect^34–37^. As our proposed stretch sensor is thin and attachable, we utilized it to qualitatively analyze the displacement change of the spine during bending or stretching, by fixing it at the positions along the spinous process joints, including cervical, thoracic, and lumbar spine (Fig. 3g) in the sagittal plane. In addition, an inclinometer based on acceleration sensing (Supplementary Fig. 12) is also employed as a comparison. Testing results are shown in Fig. 3h–n. The stretch sensor is fixed respectively along the spine on points of C1 and C7 (Fig. 3h), L1 and C7 (Fig. 3i), and S1–L1 (Fig. 3j). The inclinometer is placed right in the middle position. Taking the cervical vertebra test as an example (Fig. 3h), the subject made a nod with mild/moderate/severe amplitude, the inclinometer gave outputs ~10, 20, and 30°, whereas, the stretch sensor delivered average displacements about 13, 27 , and 38 mm, presenting an excellent correlation. Tests on thoracic and lumbar vertebra can be found in Fig. 3i, j. Simultaneously, the experimental results in different application scenes (experiment workbench/human body) and under various human postures (standing/sitting) are shown in Supplementary Fig. 7, which further demonstrates the stability of the stretch sensor.

Moreover, we performed tests on different persons. Ten volunteer participants (age 21–40 years of old; stature 159–188 cm; BMI 19.5–24.5) were recruited to participate in the study. The selection was based on the nonobese range of body mass index (BMI). We adhered to the two ends of the sensor at the positions of S1 and C7. Predictably, the displacement increased with the subject’s height (Fig. 3k). However, some exceptions occur, e.g., the 171-cm-high participant. We normalized the displacement by dividing it by the participant height, and plot it as the purple line. The normalized displacement shows that the value of the 171-cm-high participant is still smaller. We speculate that the smaller movement might be attributed to the Kyphosis appearance on the participant’s back; thus, the initial curvature on the spine results in smaller bending amplitude. In addition, participants’ bending angles are plotted as the green part, which is consistent with the normalized displacement data.

Subsequently, the device’s hysteresis is investigated and presented in Fig. 3l, m. As shown in the upper image of Fig. 3m, with the linear motor stretching, the device delivered precisely the stretching displacement (red dots), whereas, when the linear motor moves backward, the device also delivered precise releasing displacement (red pentacles). In addition, we did the verification as a function of the inclination angle (the lower image in Fig. 3m). It shows that the stretching displacement (orange circles) and releasing displacement (purple dots) coincide well. The misalignment should be due to that the subject could not ensure a perfect same bending angle during stretching and releasing. Overall, our device exhibits an ultralow hysteresis.

Figure 3n shows that under a same bending on the spine, when the two ends of the stretch sensor are placed on S1 and C7, the displacement changes largest, followed by that of sensors on L1–C7, S1–L1, and C1–C7. It indicates that the stretch sensor needs to be placed in the proper position to achieve a large value under a fixed bending angle.

### Constructing a vector stretch sensor

**Fig. 4:**
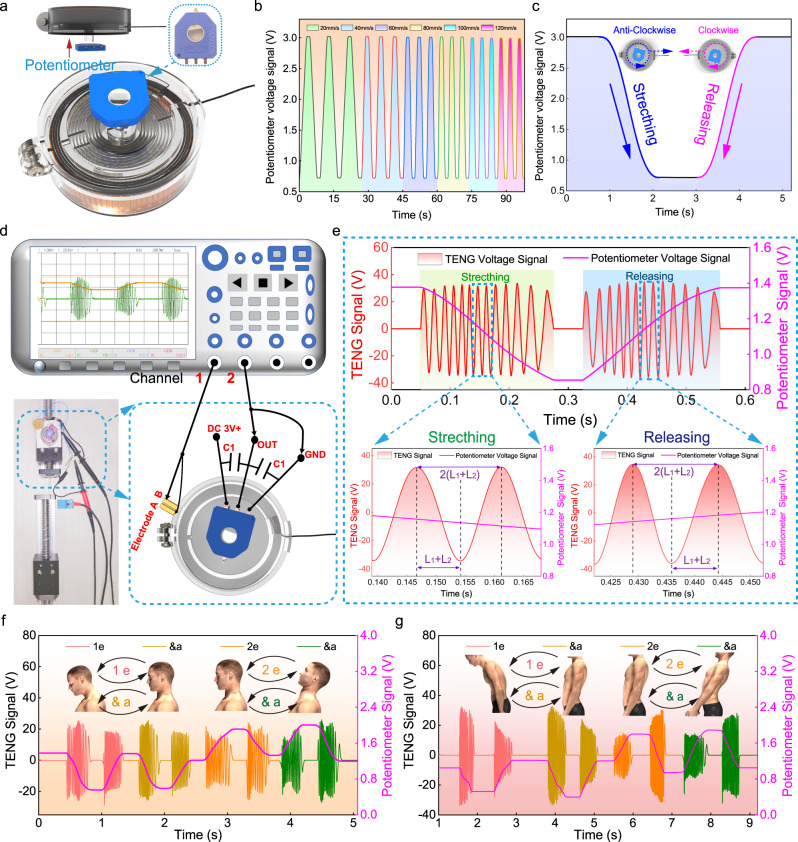
Schematic diagram and electrical characteristics of the vector stretch sensor. **a** 3D structure diagram of the vector stretch sensor. **b** The voltage output performance of the potentiometer at different stretching speeds under the same stretching displacement (60 mm), **c** Potentiometer’s output voltage curve under stretching, i.e., anticlockwise rotating, and releasing, i.e., clockwise rotating, with a rotating speed of 60 mm s^−1^. **d** Measurement circuit. **e** The measured output of the TENG device and potentiometer under stretching and releasing, the two enlarged images below indicate the displacement calculation. **f**, **g** Monitoring the signal outputs via the vector stretch sensor when the participant does some exercises, including neck/ thoracic exercise gymnastics. Source data are provided as a Source Data file.

To form a complete monitoring system, we integrated a potentiometer with high linearity (±0.2) and ultrahigh durability (1 M cycles) into the above device to construct a vector stretch sensor (the details of potentiometer performances and its various electrical parameter information are available in Supplementary Fig. 8 and Supplementary Table 1). As shown in Fig. 4a, the potentiometer is coaxially placed right above the TENG device. A tiny stainless shaft is employed to connect the TENG to the potentiometer, making two components rotate simultaneously. After applying a DC voltage source of 3 V, we measured the voltage of the potentiometer under different stretching speeds with a same displacement (60 mm) (Fig. 4b). Figure 4c illustrates the potentiometer’s voltage curve under stretching, i.e., anticlockwise rotating, and releasing, i.e., clockwise rotating, with a rotating speed of 60 mm s^−1^.

Afterward, the vector stretch sensor composed of a TENG device and potentiometer was examined. Figure 4d shows the measurement configuration, with a two-channel oscilloscope and a linear motor. Two capacitors *C1* (100 nF) are added to the two ends of the potentiometer for filtering. Outputs are simultaneously recorded by the two channels of an oscilloscope (Fig. 4e). Besides the continuous oscillation signal, a gradually increasing/decreasing voltage was obtained, from the potentiometer, exactly related to the stretching/releasing direction (Supplementary Movie 2). Additionally, we illustrate the minimum displacement, able to be measured by the stretch sensor, in the lower image of Fig. 4e, which is determined by the distance between one signal peak and one signal valley, equal to *L*_1_ + *L*_2_, as discussed above. Subsequently, we fixed the sensor on the participants’ cervical spinous process C1–C7, and thoracic spinous process L1–C7, monitoring the signal outputs when they do some setting-up exercises (Fig. 4f, g) (the test of arm and leg movement, please see Supplementary Fig. 9). Taking the cervical exercise (Fig. 4f) as an example, “1” represents lowering the head, “e” represents returning back, “&” and “a” means repeating the above process of “1” and “e”, respectively. “2” means the participant leans back the head, “e” returning back, and “&” and “a” means repeating the above process of “2” and “e”, respectively. In addition, the monitoring of thoracic exercises is plotted out in Fig. 4g. These tests show the vector stretch sensor able to serve as a wearable device for posture monitoring, physical rehabilitation training, and so on.

### Spinal and posture monitoring system

**Fig. 5:**
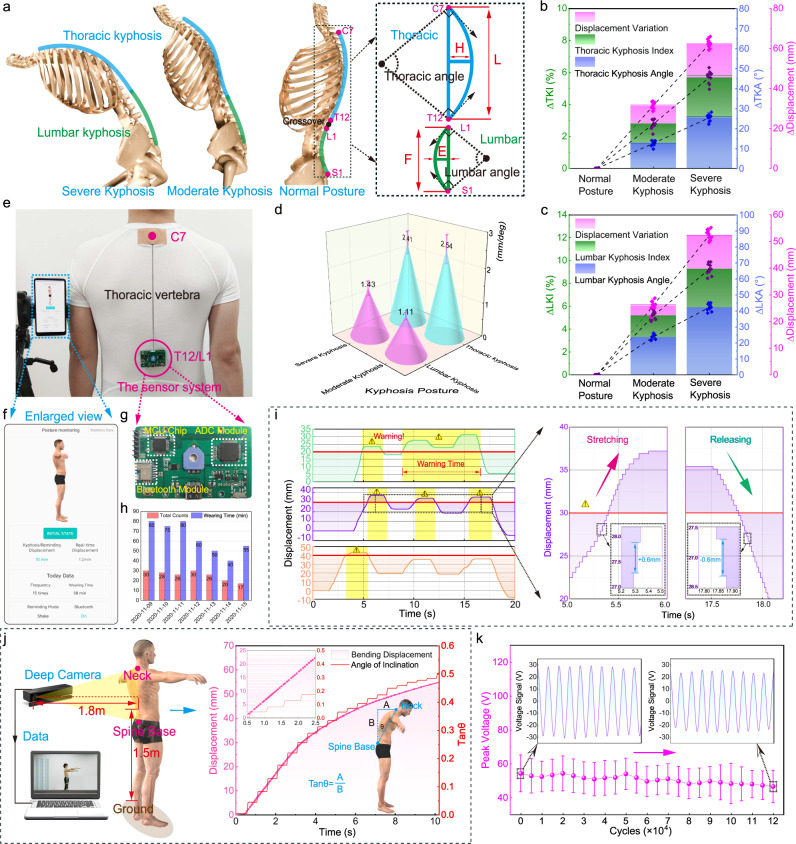
Spinal measurement and posture monitoring. **a** Three different posture states of normal posture, moderate kyphosis, and severe kyphosis, respectively, the right of the image shows the calculation method of thoracic kyphosis index (*TKI*) and lumbar spine kyphosis index (*LKI*), that is *TKI *= *H*/*L* × 100, and *LKI *= *E*/*F* × 100. **b**, **c** Indicating the changes of the thoracic/lumbar kyphosis indexes under the above-mentioned three posture states as well as the changes of thoracic/lumbar kyphosis angle and corresponding displacement. **d** The ratio of the displacement divided by the kyphosis angle to determine the fixed position of the stretch sensor. **e** Demonstration of applying the sensor system to recoding the vector displacement caused by spinal bending, and (**g**) displays the image of the integrated circuit board. **f**–**h** Mobile phone APP interface (mobile terminal) representing intraday data and weekly historical information, respectively. **i** Schematic diagram of the verification results of the sensor system feasibility analysis, and the two enlarged images represent the detailed signals of stretching and releasing, showing the gradient accuracy of +0.6 mm and −0.6 mm, respectively. **j** Synchronous continuous measurement of volunteers’ inclination angle from spine base to neck using commercial depth cameras and stretch sensor. **k** The stability and life testing of the stretch sensor. All error bars denote standard deviation based on ten replicate data under the same test condition. Source data are provided as a Source Data file.

Using the stretch sensor for real-time monitoring, our spinal motions represent a potential application that may reduce the cervical, vertebra, and back pain diseases in clinical, or decrease the number of hunchbacks. Figure 5a shows the definition of the kyphosis index (*KI*), where *L* represents the distance between C7 and T12, *H* the height of the curve, *F* the distance between T12 and L1, *E* the height. Therefore, the thoracic kyphosis index (*TKI*) can be expressed as *TKI *= *H*/*L* × 100, and the lumbar kyphosis index (*LKI*) as *LKI *= *E*/*F* × 100. The thoracic/lumbar kyphosis angle (*KA*) can be respectively calculated according to the following formulas^38,39^: *α* = 4 arctan 2*H*/*L* as well as *α* = 4 arctan 2*E*/*F*.

Figure 5b, c shows a participant’s (height = 1.75 m, weight = 70 kg, BMI ≈ 23) testing results, on the thoracic and lumbar vertebra, respectively. The *TKI* and *KA* were assessed and calculated through a traditional Flexicurve ruler (see Measurement methods: measurement of kyphosis index and kyphosis angle). Meanwhile, the stretch sensor recorded the stretching displacement. The results exhibit that the displacement increase is linearly related to the variation of the *TKI*, as the participant bends from the normal posture to the severe kyphosis.

As shown in Fig. 5d, we can define another parameter that is obtained by using the displacement dividing the kyphosis angle. The larger parameter value means under a fixed kyphosis angle variation, the displacement is larger, suggesting where our stretch sensor should be placed. Subsequently, in order to facilitate wearable and movable applications, the vector stretch sensor has been embedded into a circuit (size 4 × 6.5 cm, the detailed circuit diagram is available in Supplementary Fig. 10). Data are processed and then transmitted via Bluetooth to be displayed in an APP on a mobile phone, including intraday data and weekly summary on the spinal motion (Fig. 5f–h and Supplementary Movie 4). In order to verify the feasibility of the sensor system, we connected it to the computer through a serial port and demoed a spine motion alarm system (see Supplementary Fig. 11). According to the above analysis (Fig. 5d), we fix the sensing system between L1 and C7 on the thoracic spine of the participant. The results are shown in Fig. 5i. We set the alarm threshold value to 20, 30, and 40 mm, and the number of alarms is recorded as 2, 3, and 1 times, respectively, when the human subject completed a series of similar actions. Moreover, the enlarged images on the right of Fig. 5i show detailed signals during stretching and releasing. Each step on the stretching/releasing curve is 0.6 mm precisely. A demonstration of the posture monitoring system connected to an APP on a mobile phone can be found in Supplementary Movie 5.

Simultaneously, we utilized a commercial depth camera (Kinect 2.0, see Supplementary Fig. 12) to capture a participant’s real-time bending motions and made a comparison with the stretch sensor. The parameter tan*θ* = A/B (Fig. 5i, j) is chosen as the bending amplitude, where A, B is obtained through the customized skeleton tracking program using the Microsoft Software Development Kit (SDK, Redmond, Washington, USA, see Supplementary Fig. 13). As the human subject bends, the parameter value increased step by step, under a sampling rate of 10 Hz. In comparison, the stretch sensor output is plotted in red, exhibiting a superior continuity and precision (Fig. 5j).

Moreover, we measured the robustness of the sensor. The results indicate that the sensor can maintain stable electrical output performance after 120,000 continuous working cycles, under a given displacement of 50 mm (Fig. 5k and Supplementary Movie 6). It is worth noting that, the triboelectric layer still works. Instead, the 3D printing encapsulation box is deformed, leading to the sensor layoff (more details can be found in Supplementary Fig. 14).

## Discussion

In summary, we presented a precise, durable, and wearable stretch sensor based on triboelectric nanogenerator, and demonstrated its capability of sensing human’s joint motions and monitoring the real-time spinal bending/stretching in a sagittal plane. The test compared with the commercial inclinometer and depth camera demonstrates the feasibility and accuracy of our sensor for recording spinal motions. Experiments on over tens of participants and full-body joints confirm its ubiquity. In addition, we developed the vector stretch sensor and a spinal monitoring system, which is capable of real-time monitoring the subjects’ spinal motions, and sending out reminding wirelessly according to customized settings. Key advances involve (1) the capabilities in wearablely, conformally and real-time wireless monitoring human’s bending/stretching motions, (2) the grating-structured TENG and the peak-number-counting technique providing high precision, high repeatability, low hysteresis, and high robustness to environmental interferences, and (3) the sophisticated and scalable engineering fabrication process making the device high yield, uniform, and durable.

Notably, our proposed vector stretch sensor is capable of being mounted into the vest/kneepad for personal daily wears. However, the weak adhesion between the wears and the human skin will lead to sliding of the sensor, inducing some noise and decrease to the sensing signals. To avoid this problem, wears could be attached to the human skin with the aid of intermedia adhesive layer^40^, ensuring simultaneous and conformal bending and stretching. In addition, the device also works well for real-time monitoring of the spine and ankle motions (Supplementary Movie 7), but one more support point in the bending spot is required in order to hide the out-of-plane line. Moreover, the sensing part is based on the triboelectric nanogenerator that works actively with the self-powered ability, compared to piezoresistive or capacitive sensing devices. Generally, the stretch sensor shows outstanding durability compared with other stretchable sensors in the literature^17,41^, and the manufacturing procedure is batch fabrication, making it feasible, cost-affordable, and industry-translatable.

Overall, our proposed technology could be beneficial to reduce the risks of spinal disorders, especially for those long-term sitting in the office. In addition, it can serve as a rehabilitation brace for real-time recording of patients’ joint motions during the long recovering time after injuries. As the demand for personal healthcare monitoring and wearable sensing technologies is increasing, these precise, mass-production, wearable, and durable devices based on the triboelectric nanogenerator will impose a disruptive impact on the personal medical sensing field and therefore form a starting point for further developments of advanced wearable sensing devices.

## Methods

### Sensor fabrication

The fabrication of the sensor is mainly based on the mature technology of 3D printing and flex printed circuit board (FPCB) technology. The sensor has been made through 3D printing equipment (Object30 Prime, Stratasys, USA) to print out the sensor components, and prepare other assembly materials, such as coil springs, ropes, screws, nuts, and custom double-sided tape to assemble (the detail fabrication information is available in Supplementary Fig. 1).

### Fabrication of the Flex PCB

We used the electronic structural design software called Altium Designer 20 to draw the FPCB structural sketches and make production. The optical photograph of the FPCB is shown in Supplementary Fig. 5. Detailed fabrication procedures are revealed as follows: the pr-cleaned substrate chosen for both the rotator and stator is Polyimide (PI) with a thickness of 0.095 mm, subsequently, when the copper-clad substrate sheets are ready, which the plating thickness of copper is 0.035 mm, and then the pattern will be transferred to the substrate followed by laminate a sensitive dry layer on the top of the copper sheet, after that, the sensitive layer is exposed to patterned UV lamps via photo tools, and the nonexposure areas can be removed to reveal the copper underneath through the etching process, leaving the patterns intact. After the etching is done and unwanted copper removed, and the surface finish is applied to protect the circuit.

### Electrical measurements and materials characterization

All measurements of the stretch sensor were performed using a Keysight (Type: DSO2014A) oscilloscope, and the output voltage was supplied by an SRS (Stanford Research Systems) DS345 function generator.

### Nanostructure characterization

Field-emission electron microscopy (Hitachi SU8020) was used to characterize the nanostructures induced by the Kapton film membrane surface.

### Measurement of human body bending inclination

We used Unreal Four Engine developed by Epic Games, the SDK skeleton tracking package was customized by using blueprint and C++ to track and measure the inclination angle of human body bending in real time (Supplementary Fig. 13).

### Measurement of kyphosis index (*KI*) and kyphosis angle (*KA*)

The Kyphotic Index (*KI*) and Kyphosis angle (*KA*) will be calculated via using an architect’s Flexicurve ruler (Supplementary Fig. 12), which was molded onto the spinous process of C7 to the interspace of L5–S1. This mold was traced onto paper, and a vertical line was drawn to align the C7 spinous process and the interspace of L5–S1. After that, through drawing a horizontal line connecting the apex and the perpendicular to the thorax curve, the *KI* and corresponding *KA* will be calculated as a function of the thoracic/lumbar length and thoracic/lumbar width using standardized procedures as previously described (width/length × 100).

### Reporting summary

Further information on research design is available in the Nature Research Reporting Summary linked to this article.

## Supplementary information

Supplementary Information

Description of Additional Supplemenntary Files

Supplementary Movie 1

Supplementary Movie 2

Supplementary Movie 3

Supplementary Movie 4

Supplementary Movie 5

Supplementary Movie 6

Supplementary Movie 7

Reporting Summary

## Data Availability

All data needed to evaluate the conclusions in the paper are present in the paper and/or the Supplementary Information. Additional data related to this paper may be requested from the authors upon reasonable request. Source data are provided with this paper.

## Source data

Source Data
