# Supplementary material for: Sensing of joint and spinal bending or stretching via a retractable and wearable badge reel
Source: Nat Commun. 2021 May 19;12:2950. doi: 10.1038/s41467-021-23207-8 (PMC8136475; doi:10.1038/s41467-021-23207-8)
Supplement: Supplementary file 1 — Supplementary Information [file 41467_2021_23207_MOESM1_ESM.pdf]

Supplementary Information

Sensing of joint and spinal bending or stretching via a retractable and wearable badge reel

Chengyu Li[1,2,7], Di Liu[1,3,7], Chaoqun Xu[1,2], Ziming Wang[1,3], Sheng Shu[1,3], Zhuoran Sun[4], Wei Tang[1,2,3*], & Zhong Lin Wang[1,3,5,6*]

1 CAS Center for Excellence in Nanoscience, Beijing Institute of Nanoenergy and Nanosystems, Chinese Academy of Sciences, Beijing, 100083, China;
2 Center on Nanoenergy Research, School of Physical Science & Technology, Guangxi University, Nanning 530004, China;
3 School of Nanoscience and Technology, University of Chinese Academy of Sciences, Beijing, 100049, China;
4 Department of Orthopedic, Peking University Third Hospital, Beijing 100191, China;
5 School of Materials Science and Engineering, Georgia Institute of Technology, Atlanta, GA 30332-0245, USA;
6 CUSPEA Institute of Technology, Wenzhou, Zhejiang, 325024, China;
7 These authors contributed equally: Chengyu Li, Di Liu;
* tangwei@binn.cas.cn; zhong.wang@mse.gatech.edu

Supplementary Information

Supplementary Note 1. 3
Supplementary Note 2. 6
Supplementary Note 3. 8
Supplementary Figure 1. The detailed fabrication process of the stretch sensor. 9
Supplementary Figure 2. The elastic coefficient curves of three different coil springs. 10
Supplementary Figure 3. The working mechanism of the stretch sensor, including stretching and releasing state. 11
Supplementary Figure 4. Schematic view of the charge distribution of grating-structured TENG under open-circuit conditions. 12
Supplementary Figure 5. Operating principle, FPCB optical photos, and simulation results with different electrode widths of the stretch sensor. 13
Supplementary Figure 6. The output performance of the stretch sensor at different speeds. 14
Supplementary Figure 7. Stability test of the sensor. 15
Supplementary Figure 8. Working parameters and structure of potentiometer. 16
Supplementary Figure 9. Detection of multiple joint movements via the stretch sensor. 17
Supplementary Figure 10. Circuit diagrams. 18
Supplementary Figure 11. Measurement and verification of the stretch sensor system. 19
Supplementary Figure 12. Test equipment. 20
Supplementary Figure 13. Development and test environments. 21
Supplementary Figure 14. The optical photograph of the stretch sensor after 120,000 continuous working cycles test and the SEM of Kapton surface before and after friction. 22
Supplementary Table 1. Characteristics of the potentiometer. 23
Supplementary Table 2. Simulation parameters with the electrode width of 0.3 mm. 24
Supplementary Table 3. Simulation parameters with electrode width of 0.5 mm. 24
Supplementary Table 4. Simulation parameters with electrode width of 0.7 mm. 25
Supplementary Table 5. Simulation parameters with the electrode width of 0.9 mm. 25
Supplementary Table 6. Simulated results of displacement variation under the same two open-circuit voltages signal cycle. 26
Supplementary Table 7. Simulated results of open-circuit voltages signal cycle under the same displacement of 3.6 mm. 26
Supplementary Reference 27

Supplementary Note 1.

Theoretical analysis of the operating principle for the stretch sensor in open-circuit condition.

Owing to the assumption that the thickness of the dielectric layer (Kapton) is much smaller than its width length scale, a simplified parallel-plate capacitor model without consideration of edge effect can be reasonably introduced in which any overlapped region between the freestanding electrode and the electrodes A and B. Simultaneously, Kapton film as a triboelectric layer material is easy to get electrons, its surface will be negatively charged[1]. Therefore, we define the surface charge density of Kapton film as $-\sigma$, and the un-overlapped regions on electrode A and electrode B (regions 1 and 4 in Supplementary Fig. 4a, respectively) generate an induced charge density of σ. Considering the net charges on both electrodes A and B should be zero in open circuit condition, the induced charge density on overlapped regions (2 and 3) can be expressed as follows:

Overlapped part on electrode A (region 2):

$$\omega = -\sigma \cdot \frac{x}{L_1 - x} \tag{1}$$

Overlapped part on electrode B (region 3):

$$\omega = -\sigma \cdot \frac{L_1 - x}{x} \tag{2}$$

where x refers to the sliding distance of the freestanding electrodes, and L_1 represents the width of electrode A as well as electrode B, owing to the intermediate gap L_2 between electrode A and Electrode B is very small ($L_1 \gg L_2$), it can be ignored, in this case, we can assume the electrode's width (L_1) is approximately equal to that of the 1/2 length of the sliding TENG unit.

On the basis of the law of charge conservation and the electrical principles of electrodes in open-circuit conditions are employed to determine the charge density on different regions of the freestanding electrodes, which the formulas can be expressed as follows[2]:

Regin 2 of the freestanding electrodes:

$$\omega = \sigma + \sigma \cdot \frac{x}{L_1 - x} = \frac{\sigma \cdot L_1}{L_1 - x} \tag{3}$$

And region 3 of the freestanding electrodes:

$$\omega = \sigma + \sigma \cdot \frac{L_1 - x}{x} = \frac{\sigma \cdot L_1}{x} \tag{4}$$

According to the Gauss Theorem and combined with the charge density shown in Supplementary Fig. 4a, the electric field within the dielectric layer for region 2 and 3 can be respectively obtained as follow formulas:

$$E_{R2} = -\frac{\sigma}{\varepsilon_0 \varepsilon_r} \cdot \frac{x}{L_1 - x} \tag{5}$$

$$E_{R3} = -\frac{\sigma}{\varepsilon_0 \varepsilon_r} \cdot \frac{L_1 - x}{x} \tag{6}$$

where ε_0 and ε_r represent the permittivity of the vacuum and the relative permittivity of dielectric layer, respectively. Additionally, the potential difference between the freestanding electrode and electrode A as well as the electrode B can be respectively calculated as:

$$U_F - U_A = \frac{d \cdot \sigma}{\varepsilon_0 \varepsilon_r} \cdot \frac{x}{L_1 - x} \tag{7}$$

$$U_F - U_B = \frac{d \cdot \sigma}{\varepsilon_0 \varepsilon_r} \cdot \frac{L_1 - x}{x} \tag{8}$$

where d represents the thickness of the dielectric layer.

Owing to the freestanding electrode is made of metal, it can be considered as an equipotential body, the potential difference between the two electrodes A and B (i.e., open-circuit voltage) can be expressed as:

$$V_{OC}(x) = U_A - U_B = \frac{d \cdot \sigma}{\varepsilon_0 \varepsilon_r} \cdot \left(\frac{L_1 - x}{x} - \frac{x}{L_1 - x}\right) \tag{9}$$

However, while x approaches either 0 or L_1, the above equation S (9) is not applicable. (1) As x approaches 0, indicating that x has a small value as well as the freestanding electrode has an extremely small overlapped area with electrode B (region 3 in Supplementary Fig. 4a), which results in the value of V_{oc} obtained by the above equation goes to positive infinity. (2) Similarly, as x approaches L_1, the lapped area between the freestanding electrode and electrode A is very small, resulting in a negative infinity value of V_{oc} given via the equation (9). In the two cases discussed above, making the assumption of the parallel-plate capacitor is no longer valid. Therefore, the V_{oc} from the S(9) can only be applied to illustrate the variation trend of the freestanding electrode under sliding conditions. To calculate the V_{oc} at the situations of initial and final, the following derivation process based on electrostatics will be used.

As shown in Supplementary Fig. 4b (i), at region 1 on the left of the initial stage, the freestanding electrode is directly above electrode A, contributing to the net triboelectric charge at the contact interface is σ, while the net triboelectric charge is $-\sigma$ at region 2 on the right. Similarly, at region 2 on the right of the final stage in Supplementary Fig. 4b (ii), the freestanding electrode is precisely above the electrode B, and the net triboelectric charge at the contact interface stage is σ, as the net triboelectric charge is $-\sigma$ at region 1 on the left. According to the infinite plane model theory of uniform charging, the electric potential of electrode A and B with the infinitely position as a zero-potential reference point can be calculated separately through the following formulas:

$$U_A = \frac{d \cdot \sigma}{\varepsilon_0 \varepsilon_r} \tag{10}$$

$$U_B = -\frac{d \cdot \sigma}{\varepsilon_0 \varepsilon_r} \tag{11}$$

thus, the V_{oc} at the initial and final position can be respectively revealed by the following equations:

$$V_{OC}(0)\big|_{initial} = U_A - U_B = 2 \cdot \frac{d \cdot \sigma}{\varepsilon_0 \varepsilon_r} \tag{12}$$

$$V_{OC}(0)\big|_{final} = U_A - U_B = -2 \cdot \frac{d \cdot \sigma}{\varepsilon_0 \varepsilon_r} \tag{13}$$

Consequently, the peak-to-peak (pp) value of the V_{oc} can be expressed as follows:

$$V_{PP} = 4 \cdot \frac{d \cdot \sigma}{\varepsilon_0 \varepsilon_r} \tag{14}$$

Supplementary Note 2.

Theoretical analysis of the operating principle of the stretch sensor in short-circuit condition.

As mentioned in Note S1, Kapton film is prone to generate electrons on its surface as a friction layer material. Therefore, the operating principle of the stretch sensor can be qualitatively accounted as follows: Electrostatic induced negative charges are accumulated on electrode A, while the electrode B is positively charged with the identical amount at the initial stage. As the external mechanical force, the rope in the sensor is stretched (clockwise) or contracted (anti-clockwise), bringing about corresponding relative rotation between the rotor and the stator. As the sliding starts, taking stretched (clockwise) as an example, free electrons will flow from electrode A to electrode B until the freestanding electrode reaches the position that is precisely above the electrode B. The charge distribution at this time is exactly opposite to the charge in the initial situation. As a result, the amount of charges in this transport process can be expressed by the following equation:

$$Q = \sigma \cdot h \cdot L_1 \tag{15}$$

Simultaneously, as the freestanding electrode continues to slide forward, the flow direction of the freestanding electrode is reversed, and they alternately overlap with two grating electrodes A and B, which will generate periodic alternating electrical signals in the external circuit. The operating mechanism of contracted (anti-clockwise) rotation is similar to that of stretch (clockwise) rotation (Supplementary Fig. 5a), except that the direction of periodic current flow is opposite.

According to the theory of variable capacitance model, assuming that a voltage of electrode A and B is V_{AB}, and a transfer charge is Q_{AB}, which the relationship between them can be given by the following equation:

$$V_{AB} = -\frac{1}{C_{AB}} \cdot Q_{AB} + V_{OC} \tag{16}$$

where C_{AB} represents the capacitance of electrode A and electrode B, which can also be considered as the series of two capacitors, that is, the capacitor formed by the freestanding electrode and electrode A as well as the capacitor formed by the freestanding electrode and electrode B:

$$C_{AB} = \frac{1}{\frac{1}{C_{FA}} + \frac{1}{C_{FB}}} \tag{17}$$

$$C_{FA} = \frac{\varepsilon_0 \varepsilon_r S(L_1 - x)}{d} \tag{18}$$

$$C_{FA} = \frac{\varepsilon_0 \varepsilon_r S(L_1 - x)}{d} \tag{19}$$

$$C_{FA} = \frac{\varepsilon_0 \varepsilon_r S(x)}{d} \tag{20}$$

where $S(L' - x)$ and $S(x)$ represent the overlapped area between the freestanding electrode and electrode A as well as between the freestanding electrode and electrode B. Subsequently, by substitute the above formulas (17), (18), and (19) into (16), and the following equation can be obtained:

$$V_{AB} = -(\frac{d \cdot L_1}{\varepsilon_0 \varepsilon_r (L_1 - x) \cdot h \cdot x}) \cdot Q_{AB} + \frac{d \cdot \sigma}{\varepsilon_0 \varepsilon_r} \cdot (\frac{L_1 - x}{x} - \frac{x}{L_1 - x}) \tag{21}$$

where h is referred to the height of the freestanding electrode as well as electrodes A and B.

In short circuit condition, indicating $V_{AB} = 0$. Thus, the charge transferred between the two electrodes A and B in short circuit condition can be expressed as follows:

$$Q = \sigma \cdot h \cdot (L_1 - 2x) \tag{22}$$

Consequently, through substituting $x = 0$ and $x = L'$ into equation (21), we can get the total charge of the freestanding electrode at the initial stage and at the final stage by the following equation:

$$Q = 2\sigma \cdot h \cdot L_1 \tag{23}$$

Supplementary Note 3.

The reason for selecting open-circuit voltage signals of the stretch sensor as a sensing signal, and explained why the FPCB with the electrode width of 0.5 mm was chosen to make the stretch sensor.

Since TENG has an important characteristic of high internal resistance, the magnitude of the open-circuit voltage (V_{OC}) is much higher than the magnitude of the short-circuit current (I_{SC})[3], which is of crucial significance for improving both the resolution and signal-to-noise ratio of the stretch sensor. Furthermore, combined with equations 22 and 16 in the short-circuit condition, it can be concluded that a larger quantity of transferred charge in short-circuit conditions gives rise to a higher magnitude of open-circuit voltage. Based on the analysis, by setting the gap between electrode A and electrode B to be 0.1 mm (the optical photos of the electrode gap are shown in Supplementary Fig. 5b, c), the increased area of tribo-surface (S) can significantly enhance the voltage output performance of the stretch sensor. Consequently, it is reasonable to utilize the open-circuit voltage as the sensing signal.

According to another important characteristic of TENG, the amount of charge transferred is related to the area of the tribo-surface. Generally speaking, for the freestanding mode grating-structured TENG, the larger the area of the tribo-surface (S), the greater the amount of charge transferred, leading to the higher magnitude of open-circuit voltage. Therefore, based on the above analysis, on the premise of not losing output voltage and being able to be used as a sensing signal, we chose an FPCB with an electrode width of 0.5 mm to make the sensing system. Certainly, the FPCB with a narrower electrode width can also meet the basic requirements. It is worth noting that all measurements in this paper were carried out using the stretch sensor with an electrode width of 0.5 mm.

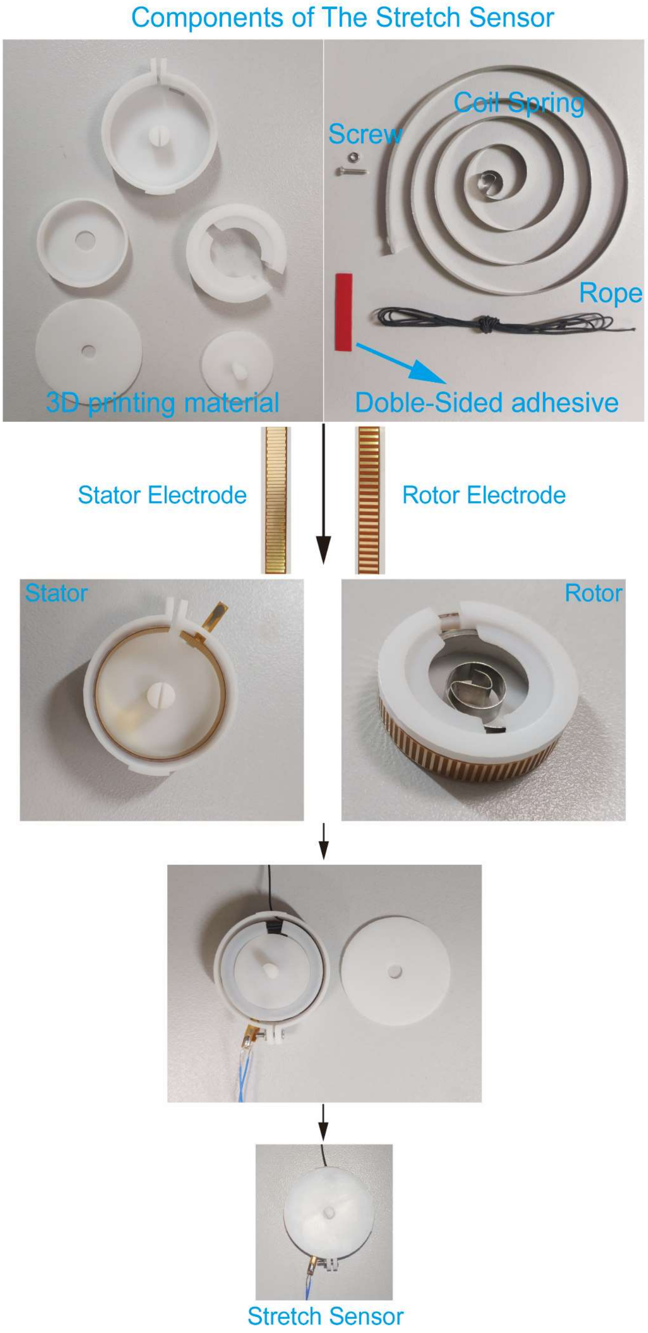

Supplementary Figure 1. The detailed fabrication process of the stretch sensor. The stretch sensor is encapsulated by 3D-printed components and other assembly materials, such as coil springs, ropes, screws, nuts, and custom double-sided tape. Simultaneously, we adhered the FPCB electrodes on the rotor and stator, respectively, and then the stretch sensor can be fabricated by integrating all the above parts.

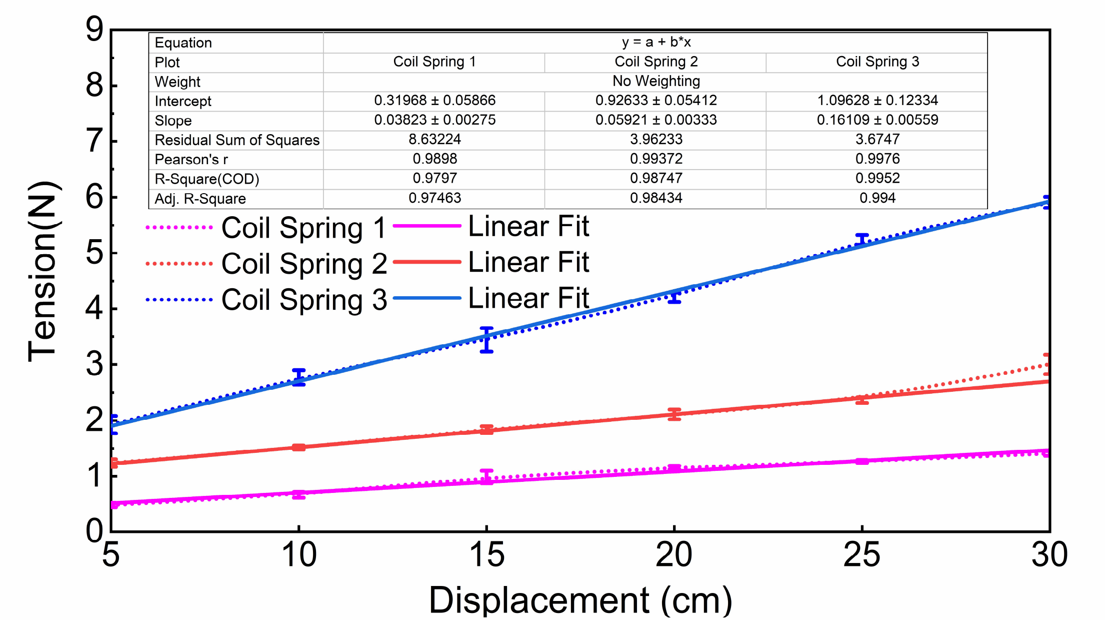

Supplementary Figure 2. The elastic coefficient curves of three different coil springs. Different slopes represent different elastic coefficients of coil springs, the greater the curve's slope, the greater the elastic potential energy. Error bars denote standard deviation based on ten replicate data.

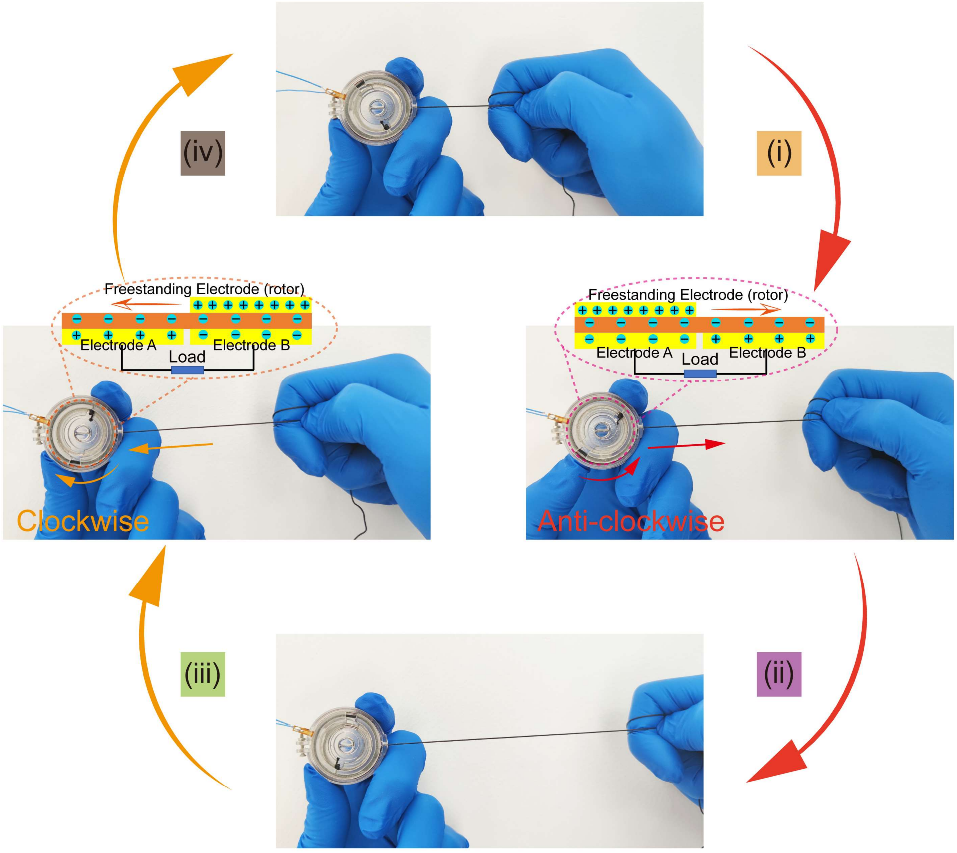

Supplementary Figure 3. The working mechanism of the stretch sensor, including stretching and releasing state. The rotor rotates anti-clockwise relative to the stator as the rope is stretched (i, ii) and clockwise as it is released (iii, iv), and insets represent the corresponding schematic diagrams under open-circuit condition. Furthermore, it should be noted that, during the release state, due to the elastic potential energy of the coil spring, the rope wrapped around the rotor will contract automatically, while the coil spring will gradually return to its original state with the potential energy decrease.

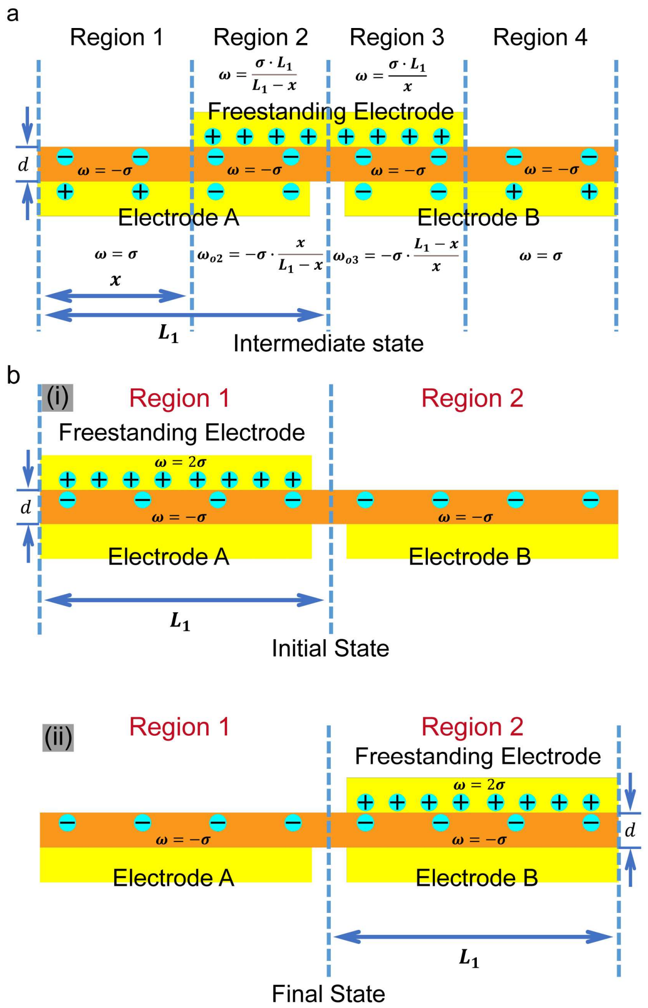

Supplementary Figure 4. Schematic view of the charge distribution of grating-structured TENG under open-circuit conditions. a Schematic illustration of a cross-sectional view of charge distribution in open-circuit at the intermediate state. **b** Schematic illustration of a cross-sectional view of charge distribution in open-circuit at the initial/final state.

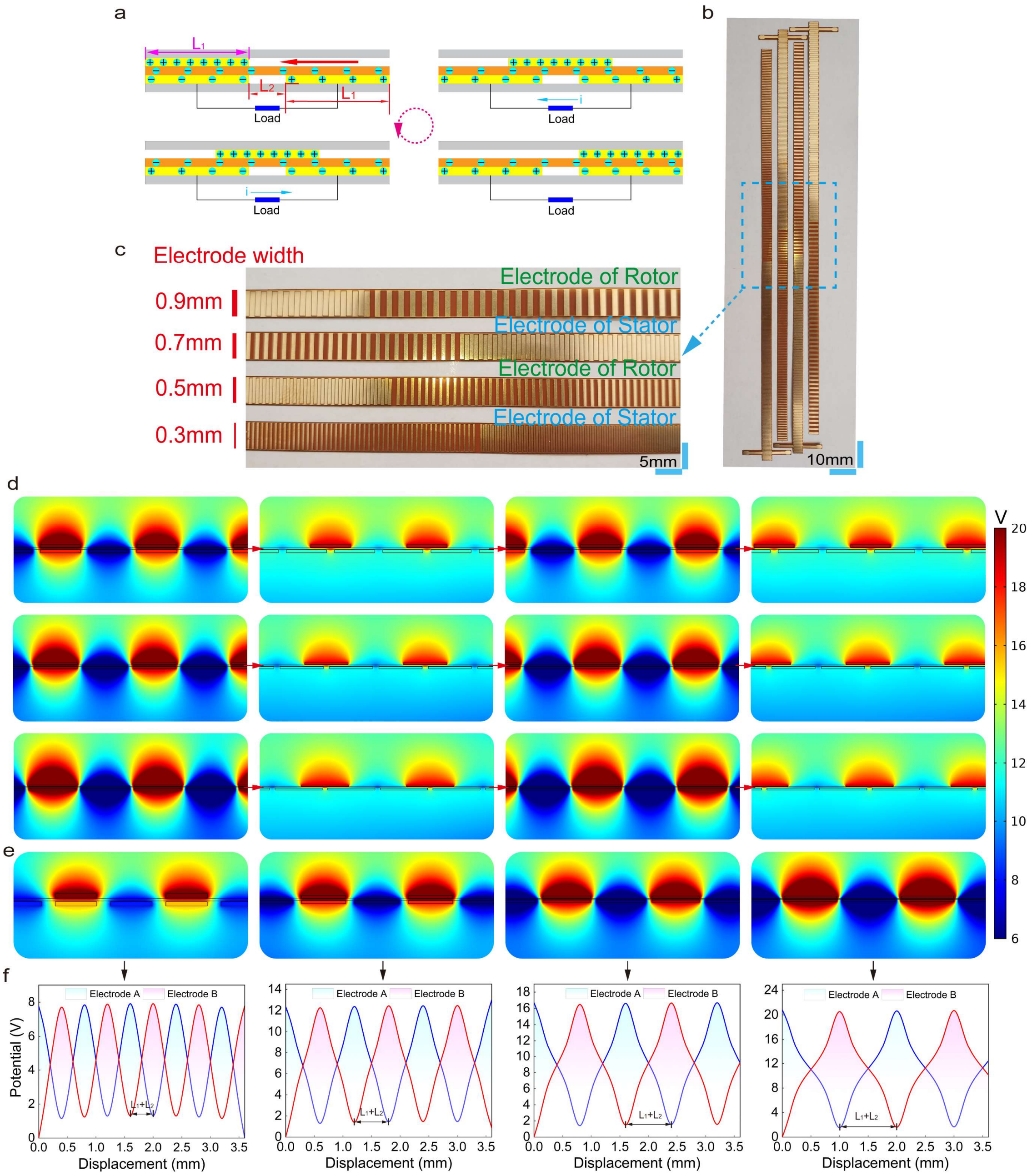

Supplementary Figure 5. Operating principle, FPCB optical photos, and simulation results with different electrode widths of the stretch sensor. a The working principle of the stretch sensor when rotating counter-clockwise, and (**b-c**) show the optical photographs of rotor and stator FPCB of different electrode widths. **d** The FEA results for the potential distribution of the stretch sensor with different electrode widths 0.5, 0.7, and 0.9 mm, respectively, in which the freestanding electrodes are sliding along a horizontal straight line in one period. **e** Simulated results for the potential distribution with different electrode widths ranging from 0.3, 0.5, 0.7 to 0.9 mm, and (**f**) its corresponding potential distribution curves.

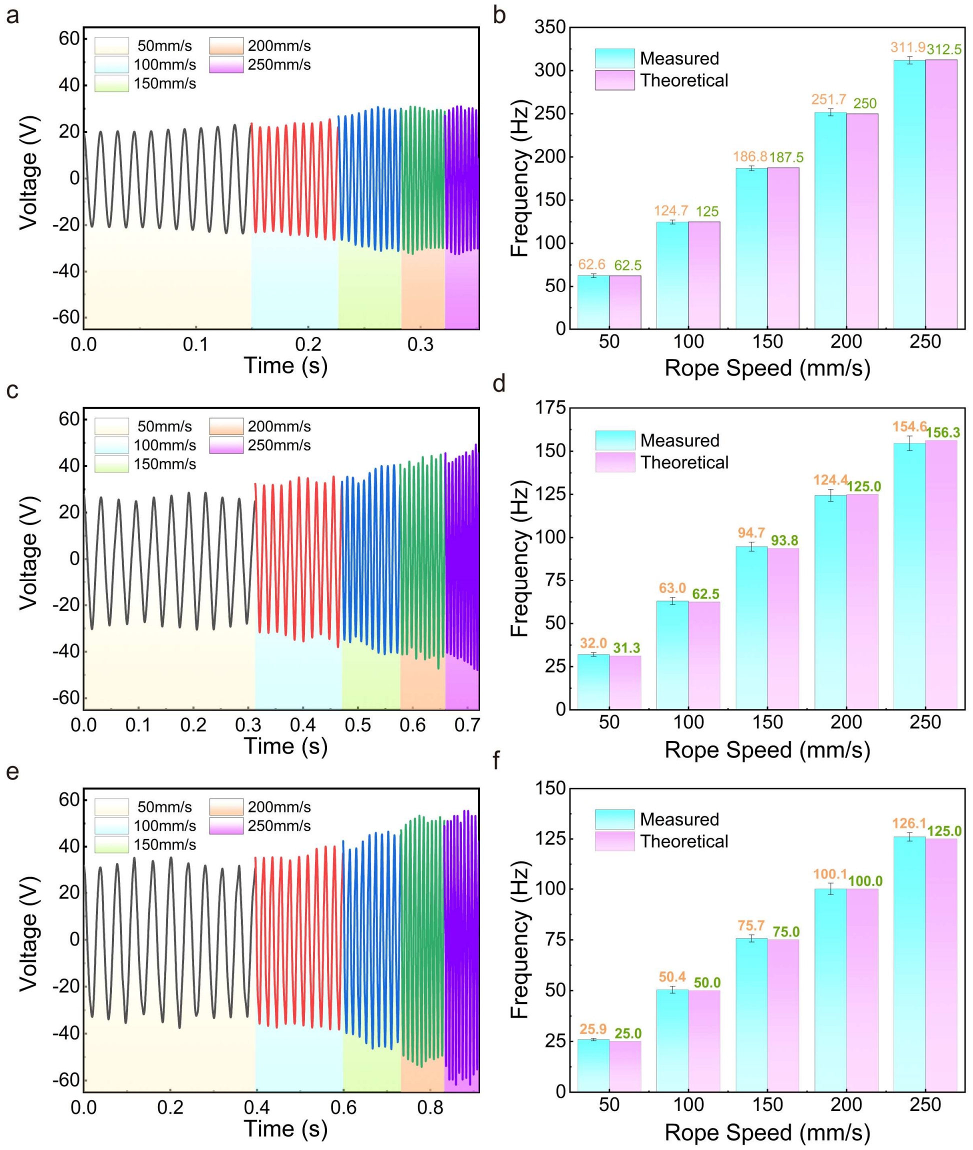

Supplementary Figure 6. The output performance of the stretch sensor at different speeds. a-f The results show electrical output performance, including voltage signals and corresponding measured/theoretical frequencies of the stretch sensors with different electrode widths of 0.3 (**a-b**), 0.7 (**c-d**), and 0.9 mm (**e-f**) at speeds from 50 to 250 mm s^{-1}. All error bars represent standard deviation based on ten replicate data under the same test condition.

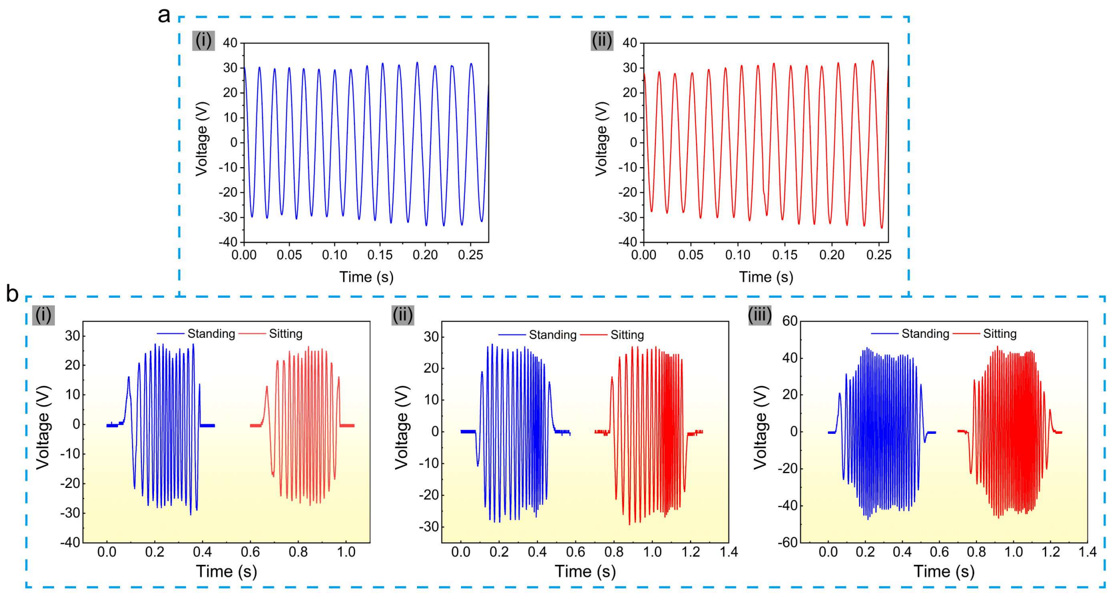

Supplementary Figure 7. Stability test of the sensor. a The results of the test, via placing the sensor on the experiment workbench and attached to the surface of the human body, respectively, indicating the signal will not be affected during human body testing. **b** The raw data results of the test when the subject is mild/moderate/severe bending while standing (blue curve) and sitting (red curve) via the sensors were fixed between spinal spines L1-C7, exhibiting the sensor system's stability.

a

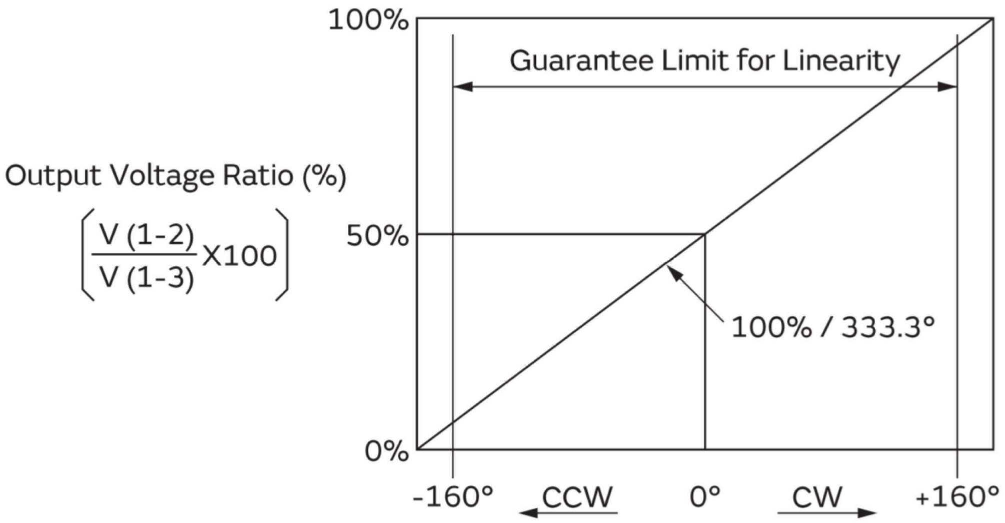

b

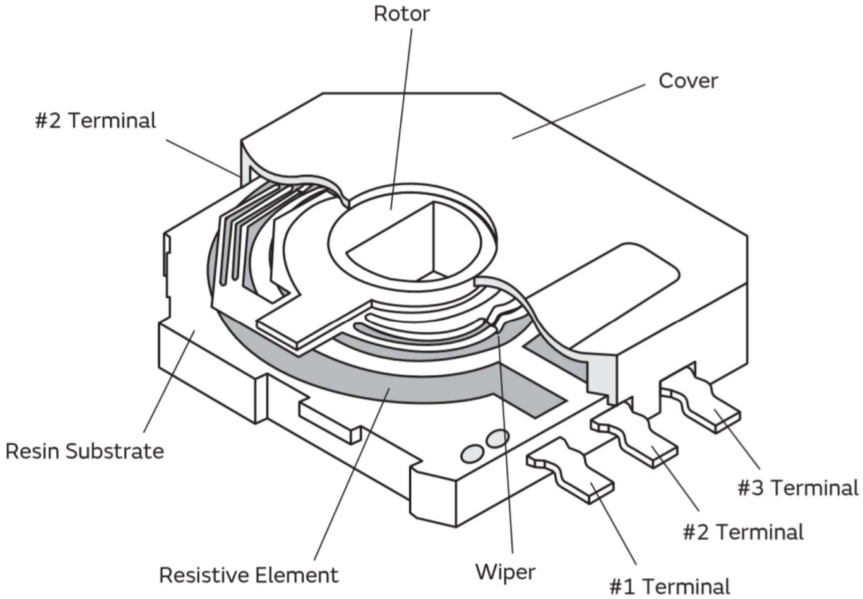

Supplementary Figure 8. Working parameters and structure of potentiometer. Depictions of the linearity interval of the potentiometer (**a**) and its construction (**b**), respectively.

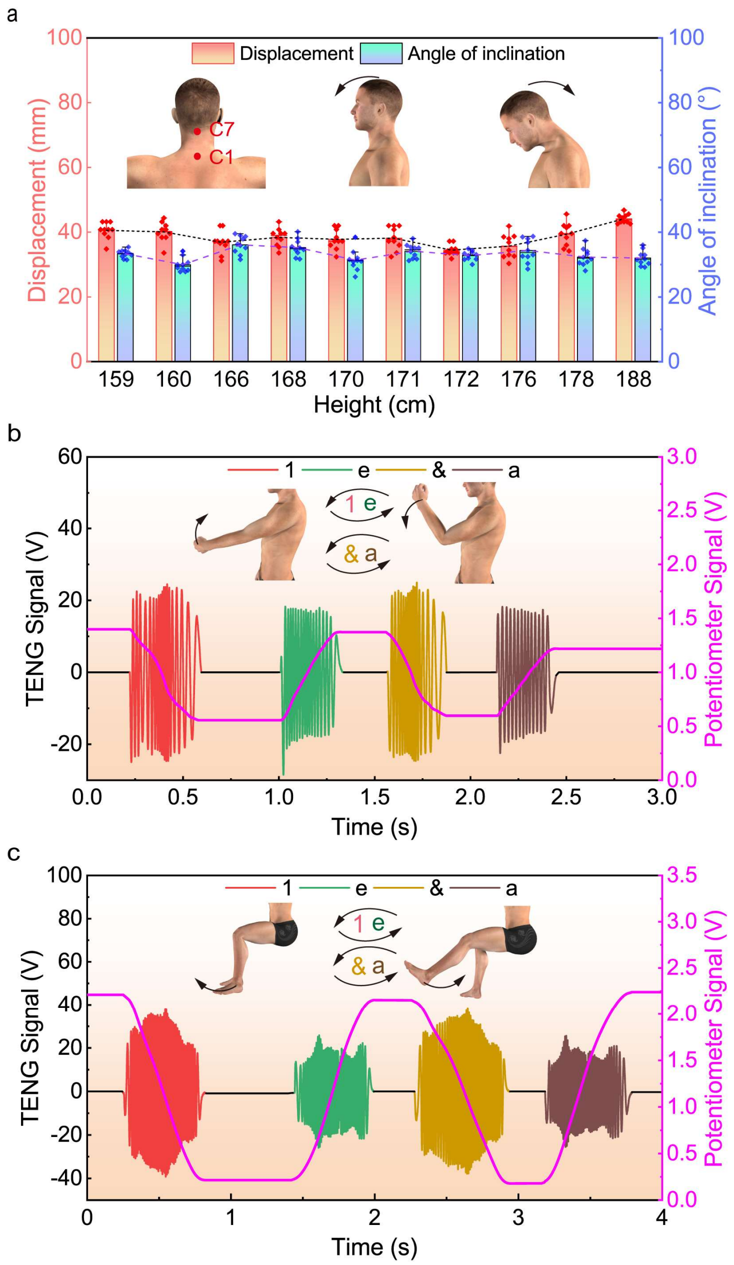

Supplementary Figure 9. Detection of multiple joint movements via the stretch sensor. a Relationship between cervical curvature angle and stretch displacement (measured by ten volunteers with different heights), in which the bending motion of the cervical spine was detected through attaching the stretch sensor to both ends of neck C1 and C7. Error bars represent standard deviation based on ten replicate data. **b-c** Monitoring the joint of elbow/knee motion via the vector stretch sensor as participants do some self-determined exercise. The vector stretch sensor is fixed on the outside of the elbow/knee, respectively, to monitor the signal outputs as the elbow/knee joint do some setting-up exercises. Taking the elbow exercise (**b**) as an example, "1" represents the elbow bending forward from the initial position, "e" represents returning to the initial position, "&" and "a" repeats the process of "1" and "e" respectively. Similarly, the monitoring of knee exercises is plotted out in **c**.

a

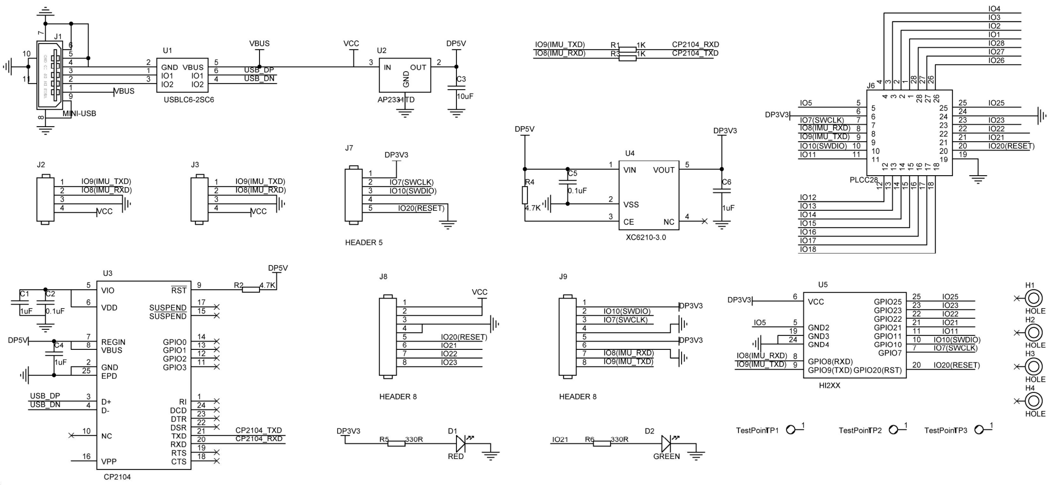

b

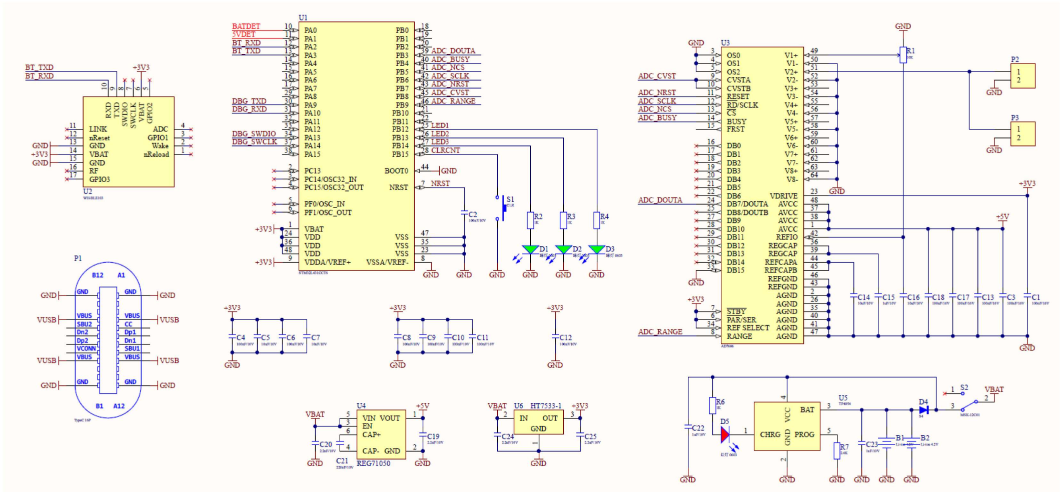

Supplementary Figure 10. Circuit diagrams. a Circuit diagrams of inertial measurement units (IMUs) for measuring the bending of the spine. **b** Circuit diagrams of the stretch sensor system for sensing of joint and spinal bending/stretching.

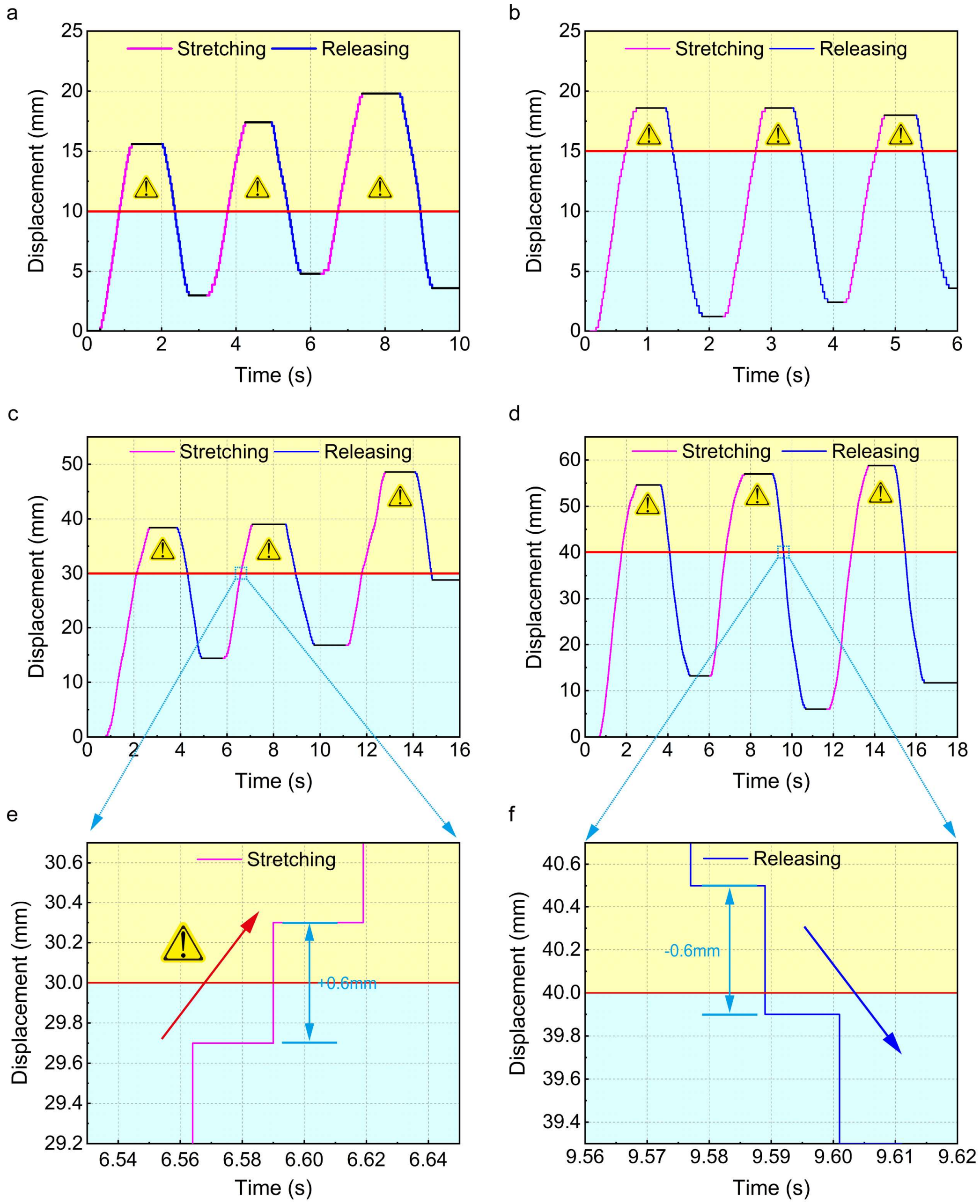

Supplementary Figure 11. Measurement and verification of the stretch sensor system. a, **b** Results of the displacement variation generated by the elbow joint swing motion measured through the stretch sensor system. The displacement alarm threshold values are set as 10 (**a**) and 15 mm (**b**), respectively, as the sensor system is fixed on the elbow and the outside of the knee joint, and the number of alarms is recorded three times. **c**, **d** Results of the displacement variation generated by the knee joint swing motion detected through the stretch sensor system. The displacement alarm threshold values are set as 30 (**c**) and 40 mm (**d**), respectively, as the sensor system is fixed on the outside of the knee joint, and the number of alarms is recorded three times. **e**, **f** The enlarged stretching/releasing diagram are provided, respectively, exhibiting the minimum resolution of the device under different stretching directions are both 0.6 mm.

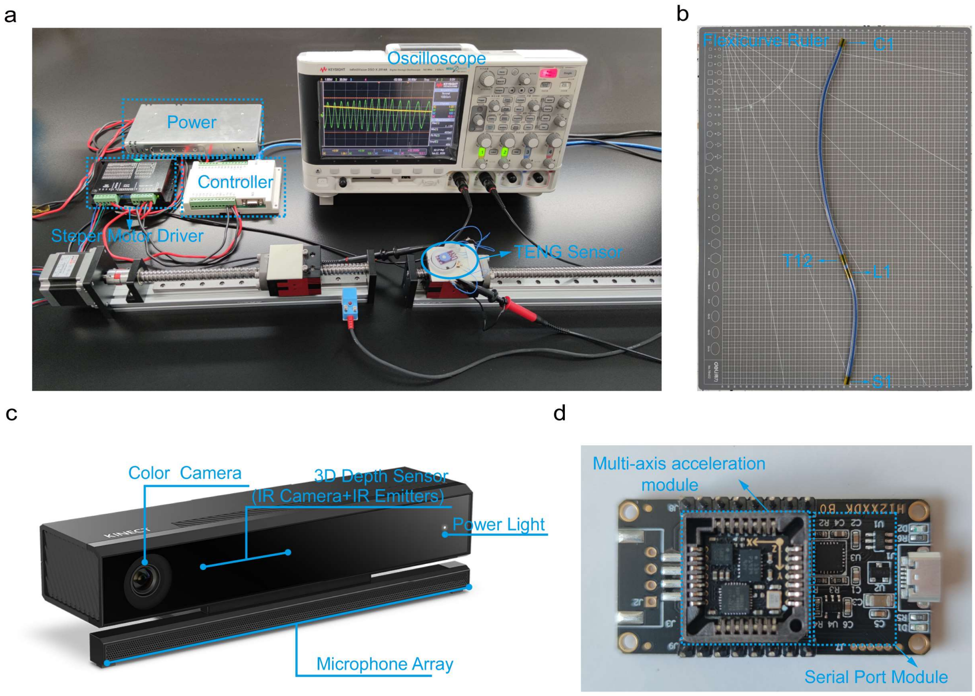

Supplementary Figure 12. Test equipment. a Linear motor test platform of the stretch sensor. **b** The optical photographs of the Flexicurve ruler. Tools for measurement of the kyphosis index (*KI*) in the thoracic and lumbar spine. **c** The optical photographs of the depth camera (Kinect for Windows SDK 2.0). **d** The optical photos of inclinometer consisting of the multi-axis acceleration sensor.

a

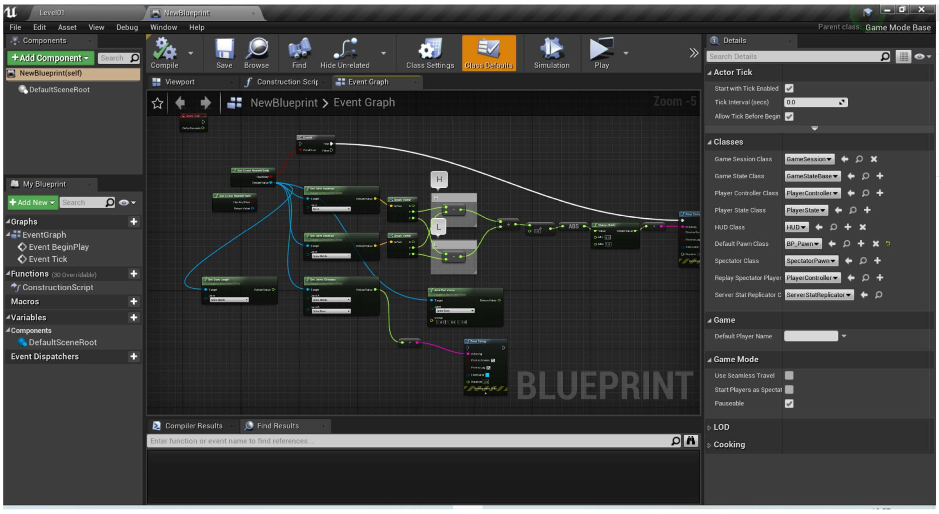

c

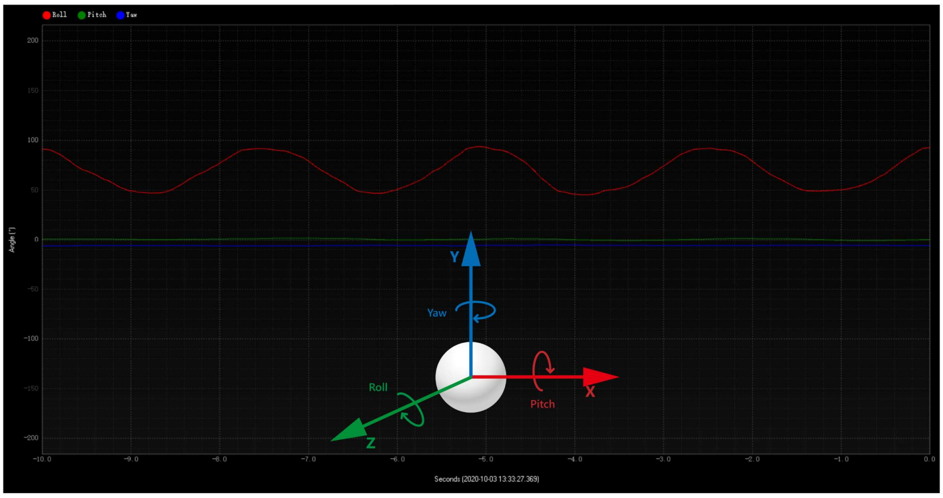

Supplementary Figure 13. Development and test environments. a Screenshot for the depth camera's development environment (Unreal Engine Four developed by Epic Games Company). **b** Illustration of the inclinometer test environment based on multi-axis acceleration module, coordinate axes roll, yaw, and pitch of different colors constitute the three-dimensional Cartesian coordinates.

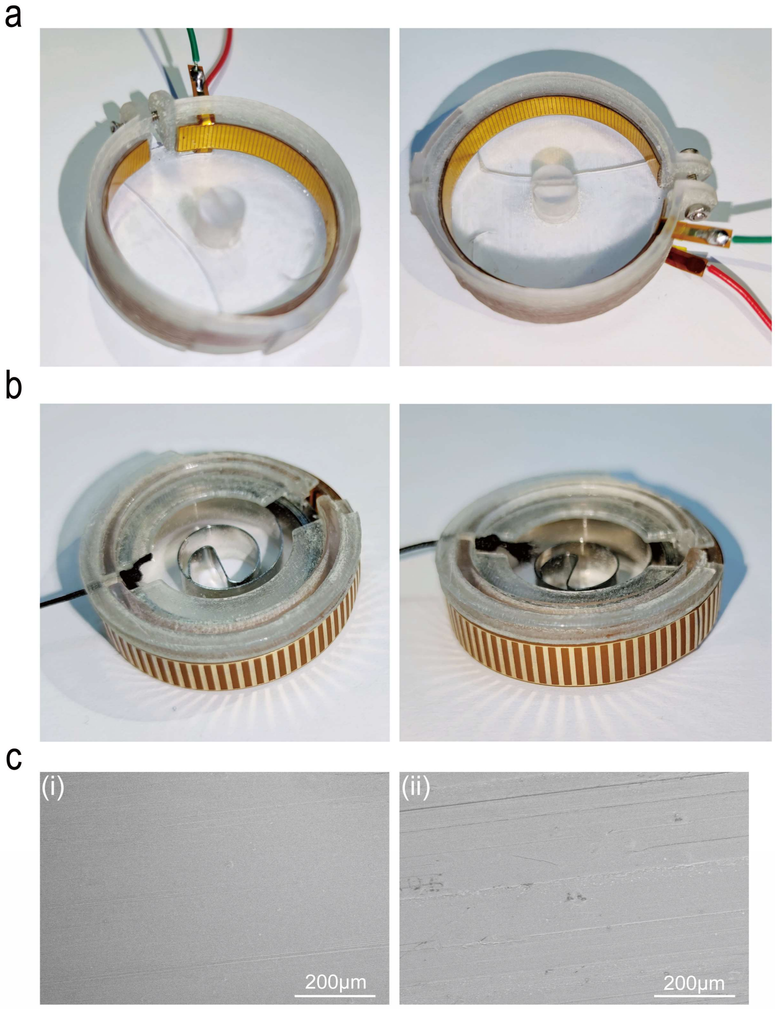

Supplementary Figure 14. The optical photograph of the stretch sensor after 120,000 continuous working cycles test and the SEM of Kapton surface before and after friction. **a, b** Optical photographs taken at different viewing angles display the wear of the friction layer of the stator (**a**) and rotor (**b**), respectively, exhibiting excellent stability and extremely high wear resistance of the stretch sensor. **c** Two images respectively represent the SEM images of surface morphology of Kapton film before (i) and after (ii) friction with the metal copper electrode.

Supplementary Table 1. Characteristics of the potentiometer

Temperature Cycle (Thermal Shock)	ΔTR :±20%<br>Linearity : ±3%
Humidity	ΔTR :±20%<br>Linearity : ±3%
Vibration	ΔTR :±10%<br>Linearity : ±3%
Shock	ΔTR :±10%<br>Linearity : ±3%
Humidity Load Life	ΔTR :±20%<br>Linearity : ±3%
High Temperature Exposure	ΔTR :±5/-30%<br>Linearity : ±3%
Low Temperature Exposure	ΔTR :±20%<br>Linearity : ±3%
Rotational Life	ΔTR :±20%<br>Linearity : ±3%

ΔTR: Total Resistance Change

Supplementary Table 2. Simulation parameters with the electrode width of 0.3 mm

Length of electrode A	0.3 mm
Length of electrode B	0.3 mm
Length of the freestanding electrodes	0.3 mm
The thickness of all of the electrodes	35 μm
Dielectric coefficient	3.5
The thickness of the dielectric layer	20 μm
The surface charge density of the dielectric layer	-7.2×10^{-7} C m^{-2}
The gap between electrodes and the dielectric layer	5 μm
The amount of displacement moved by the freestanding electrodes	3.6 mm
Step length of the movement of the freestanding electrode	0.1 mm or 0.02 mm

Supplementary Table 3. Simulation parameters with electrode width of 0.5 mm

Length of electrode A	0.5 mm
Length of electrode B	0.5 mm
Length of the freestanding electrodes	0.5 mm
The thickness of all of the electrodes	35 μm
Dielectric coefficient	3.5
The thickness of the dielectric layer	20 μm
The surface charge density of the dielectric layer	-8.7×10^{-7} C m^{-2}
The gap between electrodes and the dielectric layer	5 μm
The amount of displacement moved by the freestanding electrodes	3.6 mm
Step length of the movement of the freestanding electrode	0.1 mm or 0.02 mm

Supplementary Table 4. Simulation parameters with electrode width of 0.7 mm

Length of electrode A	0.7 mm
Length of electrode B	0.7 mm
Length of the freestanding electrodes	0.7 mm
The thickness of all of the electrodes	35 μm
Dielectric coefficient	3.5
The thickness of the dielectric layer	20 μm
The surface charge density of the dielectric layer	-9.5×10^{-7} C m^{-2}
The gap between electrodes and the dielectric layer	5 μm
The amount of displacement moved by the freestanding electrodes	3.6 mm
Step length of the movement of the freestanding electrode	0.1 mm or 0.02 mm

Supplementary Table 5. Simulation parameters with the electrode width of 0.9 mm

Length of electrode A	0.9 mm
Length of electrode B	0.9 mm
Length of the freestanding electrodes	0.9 mm
The thickness of all of the electrodes	35 μm
Dielectric coefficient	3.5
The thickness of the dielectric layer	20 μm
The surface charge density of the dielectric layer	-1.0×10^{-6} C m^{-2}
The gap between electrodes and the dielectric layer	5 μm
The amount of displacement moved by the freestanding electrodes	3.6 mm
Step length of the movement of the freestanding electrode	0.1 mm or 0.02 mm

Supplementary Table 6. Simulated results of displacement variation under the same two open-circuit voltages signal cycle

Electrode width 0.3	1.6 mm
Electrode width 0.5	2.4 mm
Electrode width 0.7	3.2 mm
Electrode width 0.9	4.0 mm

Supplementary Table 7. Simulated results of open-circuit voltages signal cycle under the same displacement of 3.6 mm

Electrode width 0.3	4.5 T
Electrode width 0.5	3.0 T
Electrode width 0.7	2.25 T
Electrode width 0.9	1.75 T

Supplementary References

1. Zou, H. et al. Quantifying the triboelectric series. *Nat. Commu.* **10**, 1427 (2019).
2. Zhu, G., Chen, J., Zhang, T., Jing, Q. & Wang, Z. L. Radial-arrayed rotary electrification for high performance triboelectric generator. *Nat. Commun.* **5**, 3426 (2014).
3. Saadatnia, Z., Esmailzadeh, E. & Naguib, H.E. High Performance Triboelectric Nanogenerator by Hot Embossing on Self-Assembled Micro-Particles. *Adv. Eng. Mater.* **21**, 1700957 (2019).
