# Supplementary material for: Sensing of joint and spinal bending or stretching via a retractable and wearable badge reel
Source: Nat Commun. 2021 May 19;12:2950. doi: 10.1038/s41467-021-23207-8 (PMC8136475; doi:10.1038/s41467-021-23207-8)
Supplement: Supplementary file 2 — Description of Additional Supplemenntary Files [file 41467_2021_23207_MOESM2_ESM.pdf]

Description of Additional Supplementary Files

Supplementary Movie 1. The simulation movie of the potential distribution during sliding.

Supplementary Movie 2. The test of the stretch sensor at different linear velocity.

Supplementary Movie 3. The measurement of knee and elbow motions.

Supplementary Movie 4. Real time monitoring of spinal bending.

Supplementary Movie 5. Posture monitoring system.

Supplementary Movie 6. Lifetime performance test of the stretch sensor.

Supplementary Movie 7. Real time monitoring system for elbow and knee motions.
